# Nanopore Detection Using Supercharged Polypeptide Molecular Carriers

**DOI:** 10.1021/jacs.2c13465

**Published:** 2023-03-10

**Authors:** Xiaoyi Wang, Tina-Marie Thomas, Ren Ren, Yu Zhou, Peng Zhang, Jingjing Li, Shenglin Cai, Kai Liu, Aleksandar P. Ivanov, Andreas Herrmann, Joshua B. Edel

**Affiliations:** † Department of Chemistry, Imperial College London, Molecular Science Research Hub, London W12 0BZ, U.K.; ‡ DWI − Leibniz Institute for Interactive Materials, Forckenbeckstr. 50, 52056 Aachen, Germany; § Institute of Technical and Macromolecular Chemistry, RWTH Aachen University, Worringerweg 1, 52074 Aachen, Germany; ∥ State Key Laboratory of Rare Earth Resource Utilization, Changchun Institute of Applied Chemistry, Chinese Academy of Sciences, Changchun 130022, China; ⊥ Engineering Research Center of Advanced Rare Earth Materials, (Ministry of Education), Department of Chemistry, Tsinghua University, Beijing 100084, China; # Department of Metabolism, Digestion and Reproduction, Imperial College London, London W12 0NN, U.K.

## Abstract

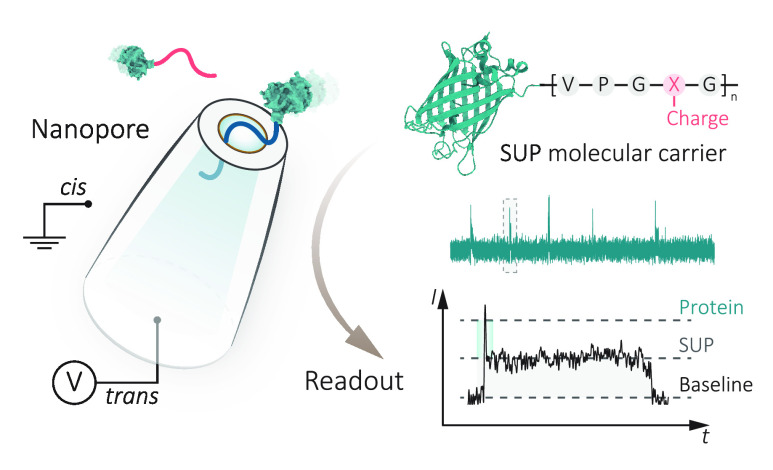

The analysis at the single-molecule level of proteins and their interactions can provide critical information for understanding biological processes and diseases, particularly for proteins present in biological samples with low copy numbers. Nanopore sensing is an analytical technique that allows label-free detection of single proteins in solution and is ideally suited to applications, such as studying protein–protein interactions, biomarker screening, drug discovery, and even protein sequencing. However, given the current spatiotemporal limitations in protein nanopore sensing, challenges remain in controlling protein translocation through a nanopore and relating protein structures and functions with nanopore readouts. Here, we demonstrate that supercharged unstructured polypeptides (SUPs) can be genetically fused with proteins of interest and used as molecular carriers to facilitate nanopore detection of proteins. We show that cationic SUPs can substantially slow down the translocation of target proteins due to their electrostatic interactions with the nanopore surface. This approach enables the differentiation of individual proteins with different sizes and shapes via characteristic subpeaks in the nanopore current, thus facilitating a viable route to use polypeptide molecular carriers to control molecular transport and as a potential system to study protein–protein interactions at the single-molecule level.

## Introduction

Proteomics analysis of a particular biological context offers unique insights into understanding the complex processes that influence human biology.^1^ By mapping the total proteins both spatially and temporally, these analyses have the potential to reveal disease-causing pathways, further discovering potential drug targets as well as diagnostic biomarkers.^2^ Currently, proteomics study heavily relies on the use of mass spectrometry (MS) to identify the sequences of digested peptide fragments.^3^ However, this technique typically requires about a billion copies of a protein for reliable characterization, thus rendering the technique unsuitable for proteins with low copy numbers. In contrast, nanopore-based analyzers provide a low-cost and high-throughput platform for real-time analysis of unlabeled single biomolecules.^4−6^ In such measurements, individual molecules are driven through a nanopore under an electric field, partially blocking the ionic current across the pore. This label-free method has enabled the straightforward characterization of biopolymers, such as nucleic acids and proteins, by analyzing their current blockade (Δ*I*_b_) caused by ion exclusion. Over the past two decades, a variety of nanopore biosensors have shown substantial progress in single-molecule biosensing, including direct DNA sequencing.^7^

Among many factors that contribute to the success of nanopore-based DNA sequencing, precise control over the target molecule transport through the nanopore is crucial.^8^ Turning now to proteins and proteomes, however, this step remains challenging as it is not always clear how to effectively transport protein through the pore.^9,10^ Unlike nucleic acids, protein molecules typically fold into three-dimensional structures with heterogeneous charge on their surface, resulting in complex translocation signals. Second, protein structures are not strictly static but conformationally flexible, further complicating the measurement and analysis process.^11,12^ Furthermore, proteins typically translocate faster than timescales that can be detected, causing a low capture rate biasing the data, especially for smaller proteins.^13^ Although this temporal limitation can be somewhat improved using high-bandwidth amplifiers,^14^ it, in turn, demands substantial optimization of nanopore noise and geometry, which is often exceptionally challenging. Taken together, controlling protein transport with high spatiotemporal resolution is, therefore, a key to developing an efficient nanopore-based protein analyzer that has the potential to facilitate single-molecule protein sequencing.^15,16^

Several strategies to date have been developed for a nanopore-based protein sequencer, including molecular carriers.^17−22^ A carrier contains a sufficiently charged body such as DNA,^21−23^ charged peptides,^17,24^ or nanoparticles^25,26^ that are easily transported through a nanopore and can act as a recognition site for protein binding. The application of carriers allows us to selectively “fish out” specific targets in complex solutions. The binding of a specific protein can be detected in the difference between signals produced by the protein carrier complex and the carrier on its own. In the former case, the presence of the protein usually induces a signature subpeak superimposed on the signal.^21^ Another advantage of this strategy is slowing down and controlling the protein translocation using carriers as they are translocated through the pore on a much longer timescale. On the basis of this concept, Yan et al.^19^ and Brinkerhoff et al.^20^ reported their initial attempt at protein nanopore sequencing inspired by the DNA nanopore sequencer, where the target peptides were controlled by conjugation with a carrier DNA molecule. The DNA carrier that was precisely motored by a translocase could pull the peptide through the nanopore in single amino acid steps, whereby the sequential difference of analyzed peptides was successfully resolved. Despite these promising results, this approach still relies on the complex DNA–peptide linkage and is limited to very short peptides.

**Figure 1:**
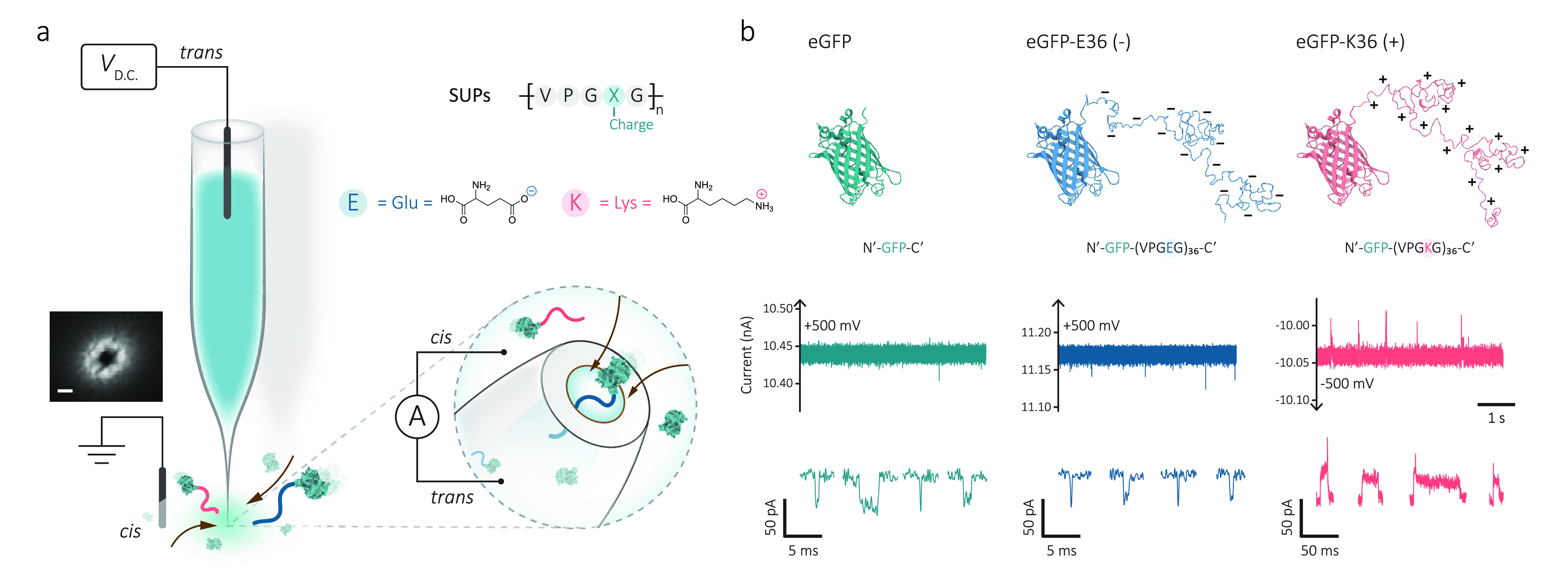
Single-molecule nanopore sensing of proteins carried by supercharged unstructured polypeptides (SUPs). (a) Schematic of the experimental setup of single-protein detection using nanopipettes. An SEM image of a typical 13 nm nanopore formed at the tip of a quartz nanopipette (scale bar: 20 nm). An Ag/AgCl working electrode was inserted into the nanopipette (*trans* chamber), and the other Ag/AgCl reference electrode was fixed in the external bath, where proteins were placed (*cis* chamber). A positive voltage to the *trans* chamber was applied to capture anionic proteins from the *cis* chamber, whereas a reversed voltage was applied for cationic proteins, as shown in the schematic. The SUP is an elastin-like polypeptide with a repetitive sequence of VPGXG, where X is a variable amino acid for modular charge or hydrophobicity. X can be glutamic acid (E) or lysine (K) for the design of anionic or cationic SUPs. (b) Structural illustration of enhanced green fluorescent protein (eGFP, 28.6 kDa, pI of 5.58), anionic eGFP-E36 (46.6 kDa, pI of 4.57), and cationic eGFP-K36 (46.6 kDa, pI of 9.83). Representative ionic current traces and typical translocation events (filtered at 5 kHz for visualization) in 1 M KCl, 10 mM Tris-EDTA, pH 8.0 buffer for eGFP (5 nM, +500 mV), eGFP-E36 (5 nM, +500 mV), and eGFP-K36 (5 nM, −500 mV) were shown, respectively. Data were recorded at 1 MHz and further processed with a 10 kHz low-pass filter for statistics.

To address these challenges, we have designed a pure protein solution that uses supercharged unstructured polypeptides (SUPs) as the carrier to precisely control the nanopore transport of whole proteins. The SUPs we used are derived from the consensus sequence of tropoelastin and have a repetitive sequence of (VPGXG)*_n_*, where X represents a variable amino acid (Figure 1a).^27^ By introducing different charged amino acids at the X position, the SUPs can be tuned to be uniformly positively or negatively charged with a high net charge.^28^ These programmable SUPs can be genetically fused with the proteins of interest without affecting their native structures and functions.^29^ Furthermore, SUPs can enhance the stability of proteins in solution^30^ to facilitate nanopore measurements, as the experiments are typically performed at relatively high salt concentrations. Much like traditional DNA carriers, the high-density charge on the fused SUPs enhances the electrokinetic driving force of the target protein as it translocates through the pore, thus dramatically improving the efficiency and resolution of the measuring system.

In principle, folded target proteins can be distinguished from linear unfolded SUPs by the extent of nanopore current blockade. We start by comparing different variants of a model protein, enhanced green fluorescent protein (eGFP), with different charged SUPs attached. The translocation of the cationic variant, eGFP-K36, exhibits a substantial slowdown effect compared to native and anionic variants. Having explored that this effect depends on the charge number of the SUPs, it is likely to stem from the electrostatic interactions between the SUPs and the nanopore.^31^ We then demonstrate the possibility of using cationic SUPs to accurately identify proteins on the basis of size and shape. Our approach also provides a route to study protein–protein interactions at the single-molecule level. The ability to perform such studies with high spatiotemporal resolution demonstrates the potential of using SUPs for precise control over proteins through nanopores and for future protein fingerprinting and single-molecule proteomics.

## Results and Discussion

### Design and Characterization of Supercharged Unstructured Polypeptides

Supercharged unstructured polypeptides (SUPs) share structural characteristics with natural elastin, such as tropoelastin found in the extracellular matrix of vertebrate cells.^29,32^ The general structure of SUPs is (VPGXG)*_n_*, where its monomeric pentapeptide unit is Val-Pro-Gly-X-Gly (Figure 1a). This repetitive sequence consists of a highly hydrophobic backbone, which ensures that the SUP does not lock into any specific conformation, and a variable residue X that determines the presence or absence of a charge. SUPs with various lengths and charges can be recombinantly expressed in the *Escherichia coli* system by engineering the repeat number *n* and the amino acid X in the gene encoding the SUP. In this work, recursive directional ligation (RDL), a stepwise procedure for oligomerization of a monomeric gene containing a defined number of repeats, was employed for the molecular cloning of the SUP genes (Figure S1).^33^ By varying the monomer length or by repeating multiple rounds of RDL, oligomeric genes with almost any desired length can be obtained. Using this method, a series of SUPs with different numbers of repeat units and thus different net charges and chain lengths, including E36, K18, K36, and K72, were produced, where E (K) denotes the glutamic acid (lysine) residue used in the X position, and the digit denotes the charge number across the polypeptide backbone. In addition, proteins of interest (POI) can be genetically fused with the unfolded SUPs via the same RDL process and expressed in their native state to obtain protein–SUP fusions. After purification by affinity chromatography and ion exchange chromatography, the fusion proteins have a purity of >90%. All expressed proteins were further characterized by both sodium dodecyl sulfate polyacrylamide gel electrophoresis (SDS-PAGE) and matrix-assisted laser desorption/ionization time-of-flight mass spectrometry (MALDI-TOF MS) or electrospray ionization time-of-flight mass spectrometry (ESI-TOF MS) (Supporting Information, Section S1, Figures S2, and S3).

### Single-Molecule Protein Sensing Using a Nanopore Platform

Nanopore experiments were performed with single-barrel quartz nanopipettes fabricated by a laser-assisted puller, as previously reported.^11^ This method yielded nanopores with a diameter of 12 ± 2 nm, as characterized by scanning electron microscopy (SEM, Figure 1a). These dimensions were in good agreement with a diameter of 10 ± 1 nm estimated using conductance measurements, given its open-pore conductance was 20.0 ± 1.6 nS (*n* = 20) in 1 M KCl, 10 mM Tris-EDTA pH 8.0 buffer (Supporting Information, Figure S4). As shown in Figure 1a, protein analytes were introduced outside the pipette with a ground/reference Ag/AgCl electrode (*cis* chamber), and a patch Ag/AgCl electrode was inserted into the pipette with only buffer solution (*trans* chamber). Ionic current traces were recorded using a high-bandwidth amplifier (Chimera Instruments, VC100) with a sampling rate of 1 MHz and a low-pass filter of 10 kHz, unless otherwise stated. A plot of the power spectral density (PSD) shows typical noise levels expected for such measurements (Supporting Information, Figure S5).

**Figure 2:**
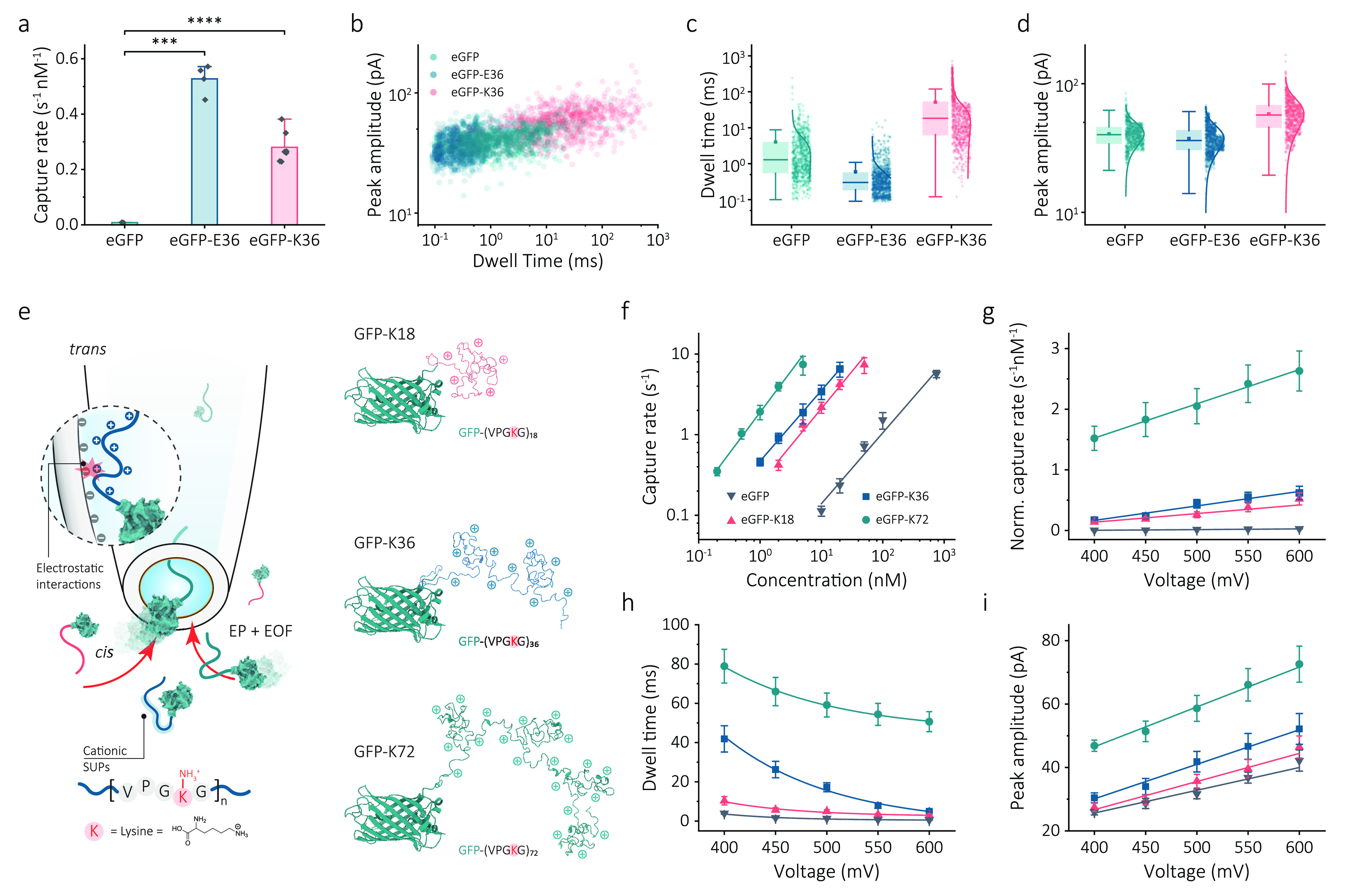
Charge- and length-dependence of the nanopore translocation for eGFP-SUPs. (a) Normalized capture rate (event frequency/concentration) for eGFP, eGFP-E36, and eGFP-K36 at the same voltage magnitude (500 mV). T scores were used to test the statistical significance between eGFP and eGFP-SUPs; ***P < 0.001 and ****P < 0.0001. (b) Scatter plots of peak amplitude versus dwell time for eGFP (*n* = 845), eGFP-E36 (n = 1074), and eGFP-K36 (*n* = 871) at 500 mV. Box and whisker plots of (c) dwell time and (d) peak amplitude for eGFP, eGFP-E36, and eGFP-K36, along with associated statistics. (e) Schematic of the cationic eGFP-SUPs translocated through a nanopore. Lysine is the key compound in the repeated sequence (VPGKG)*_n_*, where *n* represents the net charge of the SUP. Application of a negative bias to the *trans* chamber captures cationic eGFP-SUPs by both electrophoretic and electro-osmotic flows. A series of eGFP-SUPs with different charges and chain lengths (K18, K36, and K72) were shown. Ionic current was recorded in 1 M KCl, 10 mM Tris-EDTA pH 8.0 buffer. Data were sampled at 1 MHz and low-pass filtered at 10 kHz. (f) Concentration dependence of capture rate for eGFP, eGFP-K18, eGFP-K36, and eGFP-72 at −500 mV. Voltage dependence of (g) normalized capture rate, (h) dwell time, and (i) peak amplitude for eGFP, eGFP-K18, eGFP-K36, and eGFP-72. The error bars represent one standard deviation of at least three independent experimental repeats.

To validate how the SUP carrier influences protein transport through a nanopore, we first used eGFP as a model protein since it has a stiff β-barrel structure and is relatively small (27 kDa) that is not easily resolved using solid-state nanopores^34,35^ and compared the transport properties of native eGFP, anionic eGFP-E36, and cationic eGFP-K36. Under the application of an electric field, protein transport through a nanopore is subjected to the resultant cooperation and competition from diffusion, electrophoretic (EP), and electro-osmotic (EO) flow.^9^ In our configuration, we used relatively high ionic strengths (1 M KCl) to suppress the EO flow and to maximize the signal-to-noise ratio (SNR) of the nanopore measurements without disrupting the native structure of the proteins. At such conditions, protein molecules are captured and transported by the local electric field around the nanopore.^36^ As a result, the translocation of a single protein can generate a current blockade (Δ*I*_b_), which is directly proportional to the excluded ionic volume (Λ) and can be estimated as Δ*I*_b_ ≈ σψΛ/*H*_eff_^2^ (1). In this equation, σ is the solution conductivity, ψ is the applied potential difference, and *H*_eff_ is the effective length of the nanopore.^11,12^ The comparison of the translocation properties for different eGFP variants (eGFP, eGFP-E36, and eGFP-K36) is shown in Figures 1b and 2a–d.

### Comparison of Native Protein and Its Charged Variants during Nanopore Translocation

Native eGFP, with an isoelectric point (pI) of 5.58, is negatively charged at pH 8.0. A positive voltage (+500 mV) was consequently applied to drive eGFP molecules translocating from the *cis* to the *trans* chamber, accompanied by ionic current blockade corresponding to individual translocation events (Figure 1b). The same positive voltage of +500 mV was used for the measurement of eGFP-E36 (pI 4.57), which has a higher negative net charge when compared to native eGFP. By contrast, the voltage was reversed (−500 mV) to transport eGFP-K36 with a pI of 9.83 as it is positively charged. The capture rate of these three eGFP variants was extracted from the distribution of interval time between two adjacent translocation events (Δ*t*) fitted with a single exponential decay (Supporting Information, Figure S6).^37^ In light of the very low capture rate for native eGFP at nanomolar concentrations (5 nM), it is time-consuming to collect sufficient translocation events for statistical analysis. Also, because the capture rate is proportional to the bulk concentration of the analyte,^4^ a much higher concentration (750 nM) was also used to improve the measurement efficiency (Supporting Information, Figure S7). The normalized capture rate (7.6 × 10^–3^ ± 0.8 × 10^–3^ s^–1^ nM^–1^) was compared with that of supercharged eGFP-E36 and eGFP-K36 in Figure 2a. When proteins translocate at a certain concentration, the capture rate mainly depends on their biophysical properties, such as diffusion coefficient, net charge, and size relative to the pore.^4^ Detection of small proteins such as eGFP often yields a lower-than-expected capture rate since it is difficult to resolve such fast translocation events with a sufficiently high SNR.^13,34^ In comparison, supercharged eGFP-E36 and eGFP-K36 exhibited 70- and 37-fold improvement in the capture efficiency, respectively, at the same magnitude of applied voltage (Figure 2a). The p-value analysis shown in Figure 2a also confirmed that these improvements could be attributed to the introduction of SUP carriers. The SUP carriers can elevate the charge density of individual eGFP molecules, thus increasing the EP driving force that proteins are subjected to during nanopore transport.

Improving spatiotemporal resolution of protein sensing is central in the nanopore community since it is directly related to the accuracy and efficiency of protein detection and, ultimately, single-molecule protein sequencing.^16^ Dwell time and peak amplitude of translocation events are two key characteristics that intuitively reflect the spatiotemporal resolution of a nanopore measurement system. Two-dimensional scatter plots of dwell time versus peak amplitude shown in Figure 2b suggest that eGFP-K36 has the highest spatiotemporal resolution among the three eGFP variants. The dwell time is closely related to the electrokinetic transport of proteins within the local electric field and interactions with the nanopore. The mean dwell time for native eGFP was found to be 1.3 ± 0.2 ms, which is consistent with a previous publication that used a similar nanopore system.^35^ By contrast, eGFP-E36 molecules translocated faster (0.3 ± 0.1 ms); however, the translocation of eGFP-K36 molecules was much slower, 18.2 ± 2.6 ms, Figure 2c, while the mean peak amplitudes for eGFP-K36 and eGFP-E36 were similar at 42 ± 3 and 36 ± 2 pA, respectively, Figure 2d. We attribute this change in dwell time to the introduction of the SUP carriers that altered the interaction between the protein molecules and the nanopore during the protein translocation.^31^ A similar slowing down of translocation events was observed for DNA molecules translocating through a biological nanopore internally engineered with positive charges.^38^ In our experimental configuration, the surface of the quartz nanopores is negatively charged at pH 8.0. Negatively charged eGFP-E36 would therefore undergo electrostatic repulsion with the nanopore walls, reducing its adhesion. In contrast, positively charged eGFP-K36 is attracted to the surface of the nanopore, substantially slowing down its translocation speed and improving the temporal resolution. This increase in dwell time improves the event detection rates and, at the same time, helps in accurately determining the amplitude of the translocation signal.^39^

### Length and Voltage Dependence upon Nanopore Translocation of Cationic Protein Variants

Having established that cationic SUPs can significantly slow down the translocations, we further investigated a series of cationic GFP-SUPs with different positive charges to confirm its mechanism (Figure 2e). eGFP-K18, eGFP-K36, and eGFP-K72 were synthesized by tuning the cycle number of RDL and characterized by gel electrophoresis and mass spectrometry in Supporting Information Figures S2 and S3. First, the dependence of the capture rate on the charges of SUP carriers was investigated (typical traces are shown in Figure S8). Previously, it has been shown that for pores larger than the analyte, the capture rate of a protein is linearly proportional to the voltage and protein concentration.^34^ As expected, this trend can be seen in Figure 2f for three different cationic eGFP-SUPs. More importantly, the capture rate increases as more positive charges are introduced, which is attributed to the increase of EP driving forces with increasing net charges. Compared with eGFP on its own, a ∼17, ∼29, and ∼136-fold capture rate increase was observed for eGFP-K18 with +18 net charges, eGFP-K36 with +36 net charges, and eGFP-K72 with +72 net charges, respectively. The voltage showed a linear dependence of normalized capture rate for eGFP, eGFP-K18, eGFP-K36, and eGFP-K72 (Figure 2g). However, the capture rate plots of eGFP and eGFP-K18 were more consistent with an exponential increase with voltage (Supporting Information, Figure S9), indicative of translocation based on a barrier-limited regime that describes the capture of short molecules under a weak EP force.^40^ As the length and charge of the SUPs increase, a more strictly linear voltage dependence is observed for both eGFP-K36 and eGFP-K72. This transition indicates the effective capture changes to a diffusion-limited regime, wherein long molecules are subjected to a strong EP force during nanopore transport.^41^ These results suggest that the introduction of more positive net charges could substantially enhance the protein capture efficiency even further.

As seen in Figure 2h, with increasing voltage, the dwell time for each cationic eGFP variant decays exponentially as the voltage reduces the energy barrier of protein translocation.^4^ This voltage dependence confirms that individual protein molecules translocate through the nanopore rather than bind and dissociate with the nanopore due to strong electrostatic interactions.^42^ Similarly, using native eGFP as a reference, the mean dwell time measured for eGFP-K18, eGFP-K36, and eGFP-K72 was 3.7-, 13.0-, and 44.7-fold slower than that of eGFP, respectively (500 mV). Provided that the length and charge of eGFP-K36 and eGFP-K72 fusions are only doubled and quadrupled compared to eGFP-K18, respectively, this increasing slowdown effect is not only due to the increase in the polypeptide contour length translocated but caused by the increasing electrostatic interactions within the nanopore.^43^ An increase in the peak amplitude was observed with the increasing SUP length, Supporting Information, Figure S10. This increase is associated with the increase in molecular weight of eGFP-SUPs, predominantly due to the increased ion exclusion inside the sensing region.^12^ The peak amplitude of each eGFP variant linearly increases with the applied voltage shown in Figure 2i, which is consistent with the relationship shown in equation (1). Having demonstrated that the performance of nanopore sensing would be much improved with increasing positive charges introduced into the SUP chain, K72 shows its potential as a molecular carrier for protein sensing with a high capture efficiency and spatiotemporal resolution.

### SUP Carriers for Improved Protein Detection

SUPs are genetically programmable, and thereby, the sign and amount of the protein charge can be flexibly tuned depending on the practical application. The introduction of SUPs can dramatically improve the capture rate and detection efficiency, irrespective of the positive or negative charge used. In this work, by utilizing the electrostatic attraction between opposite charges, cationic SUPs can slow down the protein translocation over an order of magnitude, thus offering more opportunities to improve detection accuracy. This allows for sufficient analysis of individual translocation events, further revealing structural details or binding states via subpeak analysis. This analytical method is based on the difference in the excluded volume between molecular carriers and target proteins, which has been applied in DNA-carrier-assisted protein screening.^21,23^ At the binding site, folded proteins typically exhibit a larger current blockade than linear DNA carriers, with a corresponding subpeak manifested during individual events. By comparing the peak amplitude of the different subpeaks and their fractional positions (relative position of the subpeak) along each event, this strategy can be used for multiplexed sensing of protein biomarkers at the single-molecule level in unprocessed biofluids.^44^ However, since DNA translocates relatively quickly through a nanopore, the approach typically requires the use of long DNA carriers (a few μm in length) to resolve subpeak features.

Furthermore, the subpeaks caused by the random folding of long-chain DNA carriers could interfere with the determination of protein subpeaks, increasing the probability of false positives.

Having demonstrated that cationic SUPs perform better with increasing charge, control experiments of only SUP carriers (K72) were first performed and compared with lambda-DNA, a typical dsDNA carrier used in nanopore sensing (Supporting Information Figure S11). The mean dwell time for K72 translocation was 40.1 ± 5.0 ms at an applied voltage of −500 mV. As K72 is threaded into the nanopore with a linear conformation, its average translocation speed can be estimated to be 3.6 nm ms^–1^ assuming 0.35 nm per amino acid,^45^ which is about 4000-fold slower than that of λ-DNA under the same experimental conditions (Supporting Information, Figure S12). We could also observe four distinct populations in the distribution for λ-DNA translocation. Among these populations, the first is attributed to the unfolded DNA translocation, while the rest correspond to the DNA molecules folded to different degrees, as previously reported.^46^ These complex folding states could interfere with the subpeak analysis if the carriers are bound with target proteins. In contrast, only two distinct current levels are observed in the current blockade signal, corresponding to two populations that can be identified in the scatter plot of K72 (Supporting Information, Figure S11), in accordance with its unfolded and folded states, respectively (Supporting Information, Figure S11).

**Figure 3:**
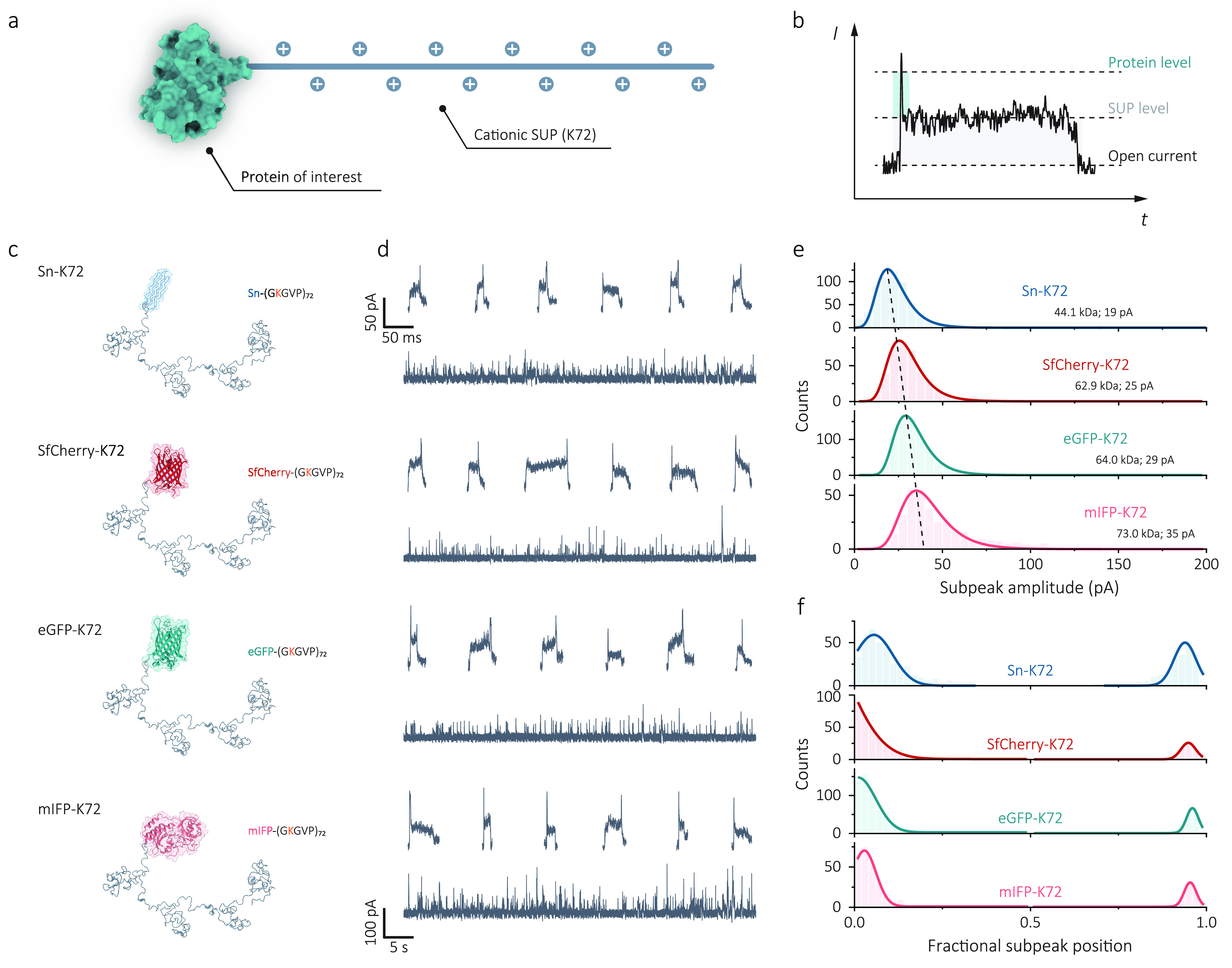
Size-dependent single-protein identification using cationic SUPs and subpeak analysis. (a) Schematic of protein detection carried by K72 carriers, where the protein of interest was attached at one end of K72. (b) Schematic illustration of a typical event of one protein–SUP molecule during nanopore translocation. The relationship between the excluded volume (Λ) and the blockade current (Δ*I*_b_) can be estimated as Λ = Δ*I*_b_*H*_eff_^2^/(σψ) (1), indicating that current blockade is proportional to the excluded volume of translocated molecules. A folded protein, therefore, has a greater excluded volume per unit length than that of an unfolded, linear SUP. Individual nanopore readouts are shown with the protein level as a higher current blockade and the SUP level as a lower current blockade but longer time residence. (c) Structures of a series of proteins with different sizes, Sn (13.0 kDa), SfCherry (20.3 kDa), eGFP (28.6 kDa), and mIFP (35.2 kDa), fused in K72 carriers. (d) Representative ionic current traces (scale bar: 100 pA; 5 s) and typical individual translocation events (scale bar: 50 pA; 80 ms) for the corresponding structures were filtered to 5 kHz for better visualization. Protein translocation was performed in 1 M KCl, 10 mM Tris-EDTA pH 8.0 buffer at −500 mV, and processed with a low-pass filter of 10 kHz. (e) Distributions of the subpeak amplitude extracted from individual translocation events for Sn-K72 (*n* = 578), SfCherry-K72 (*n* = 389), eGFP-K72 (*n* = 765), and mIFP-K72 (*n* = 341). The mean amplitude of subpeaks shows an increasing trend as the size of proteins increases. Distribution is fitted by the Gumbel function. (f) Distributions of fractional subpeak position (i.e., relative location) suggest subpeaks located at either start or end of individual events, consistent with the protein–SUP structure. Protein Data Bank (PDB) codes: Sn, 3BOI; SfCherry, 4KF4; eGFP, 2Y0G; mIFP, 5VIQ.

Proteins with different sizes and shapes were covalently extended with a cationic K72 SUP to form a protein–K72 structure and were used to demonstrate the feasibility of SUP carriers (Figure 3a). As the fusion protein is threaded into the nanopore, in principle, a two-level current signature would be expected, as illustrated in Figure 3b. The first level is consistent with the translocation of SUP carriers with a long duration comparable to that of K72 translocation without protein fusion. The second level arises at the beginning or the end of the event, corresponding to the translocation of the folded target protein with a shorter dwell time but a more significant current blockade. This increase in the current blockade is attributed to a greater volume exclusion per unit length of folded proteins than unfolded SUPs. In Figure 3c–f, we present the ability of K72 SUP carriers to effectively control and distinguish the target proteins with different sizes based on their subpeaks observed in individual translocation events. Four proteins sized from 13.0 to 35.2 kDa were compared, namely, antifreeze protein (Sn, 13.0 kDa) and three fluorescent proteins: red fluorescent protein (SfCherry, 20.3 kDa), enhanced green fluorescent protein (eGFP, 28.6 kDa), and monomeric near-infrared fluorescent protein (mIFP, 35.2 kDa), as illustrated in Figure 3c.

Representative current traces and individual events for these four protein-K72 fusions are shown in Figure 3d, in which the same low concentration of 1 nM was used in the nanopore measurement. Current–time traces revealed that the capture rate of each protein-K72 was sufficiently high for single-molecule analysis and varied little in all cases, which suggested that the SUP (K72) carrier can dominate the protein transport independent of the size and net charge of target proteins. After being processed with subpeak classification, events with a two-level signature were screened out to be ∼36, ∼38, ∼46, and ∼42% in proportion for Sn-K72, SfCherry-K72, eGFP-K72, and mIFP-K72, respectively. Given the high purity of the protein–SUP fusions, it is likely that the events without subpeaks are caused by a lack of sufficient temporal resolution. Detection could be improved further by using a higher bandwidth amplifier.^13,14^ Interestingly, the ratio of detectable subpeaks slightly increased with the molecular weight of target proteins, probably due to increased SNR and dwell time for larger proteins. This size dependence was also observed in the distribution of subpeak amplitude extracted from individual events (Figure 3e), with a well-defined single population for all proteins. The mean subpeak amplitude of these proteins exhibited a linear increase with molecular weight, which is consistent with the expectation for globular proteins (Supporting Information, Figure S13).^47^ This high sensitivity may allow for the precise identification of various proteins and recognition of interactive activities that may cause a change in volume exclusion within the nanopore. In addition, the majority of events for all four protein-K72 fusions had a single subpeak at the beginning or the end of these events, with the fractional position being 0.05 or 0.95, respectively, as shown in Figure 3f. As a comparison, a control experiment of V40-K72, which is K72 connected to an uncharged linear polypeptide (V40), was performed, where the translocation events were observed with no protein-bound-like subpeaks similar to folded proteins in previous cases (Supporting Information, Figure S14). More importantly, this subpeak recognition can also work at a near-physiological salt concentration (100 mM KCl), with two distinct current levels detected (Supporting Information, Figure S15). However, higher voltages must be used to overcome the stronger electrostatic interaction at low ionic strengths, while the nanopore is often irreversibly blocked due to this strong interaction.

**Figure 4:**
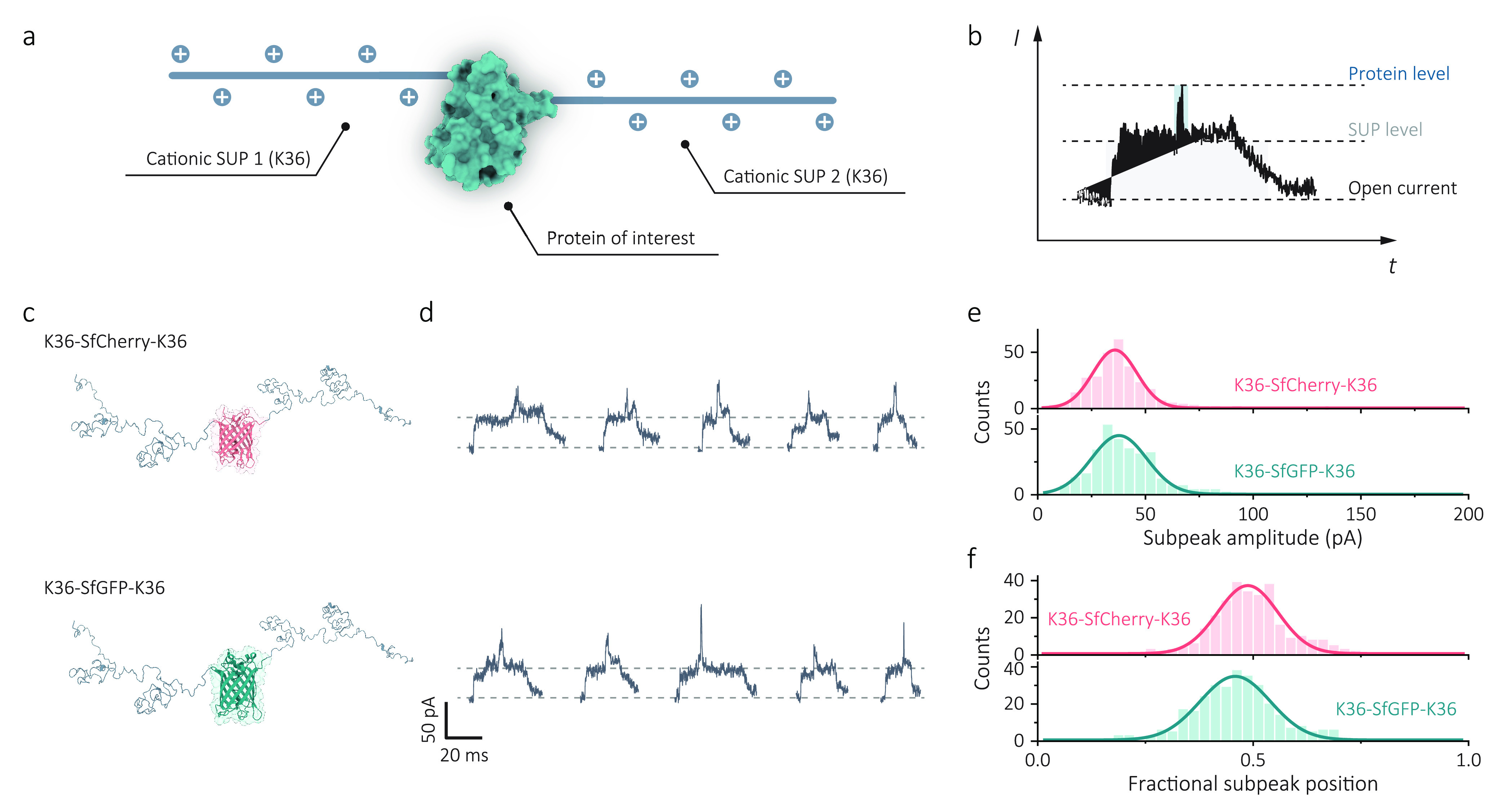
Subpeak analysis of flexible design of protein–SUP structure. (a) Schematic of the structure of K36-protein-K36, in which the net charge is equivalent to K72 carriers, but the target protein is bound at the center of the carrier. (b) An example translocation event for K36-protein-K36 shows that the subpeak is shifted to the center of the signal where the target protein is. (c) Structures of K36-SfCherry-K36 and K36-SfGFP-K36 used for single-molecule protein identification. (d) Representative translocation events (scale bar: 50 pA; 20 ms; filtered to 5 kHz) indicated that nanopore signals were consistent with the protein structures with a folded protein at the center. Nanopore experiments were performed in 1 M KCl, 10 mM Tris-EDTA pH 8.0 buffer at −500 mV with a low-pass filter of 10 kHz. (e) Distributions of the subpeak amplitude extracted from individual events for K36-SfCherry-K36 (*n* = 286) and K36-SfGFP-K36 (*n* = 307) were fitted with the Gaussian function. (f) Distributions of fractional subpeak position suggest that subpeaks are located at the center of individual events as designed in the structure.

Having demonstrated that this strategy is suitable for generic proteins regardless of their size and charge, we designed another carrier in which the target protein is located at the center to form a K36-protein-K36 fusion without changing the total charge of the SUP carrier (Figure 4a). Given that the random folding of linear biomolecules is most likely to occur at the ends of the molecules, using this structure design, false positives caused by the partial folding of carriers can be avoided to the maximum extent.^48^ Meanwhile, the addition of recognition sites at different fractional positions of a single carrier enables multiplexed high-throughput protein sensing.^21^ Example events again show a two-level current signature, but the subpeak shifts to the center, where the target protein is attached (Figure 4b). We used two common fluorescent proteins, SfCherry and SfGFP (Figure 4c), for the nanopore analysis due to their relatively small sizes and stiff structures. K36-SfCherry-K36 and K36-SfGFP-K36 displayed higher capture rates comparable to that of protein-K72 fusions at a typical concentration of 1 nM (Supporting Information, Figure S16). Processed with the similar subpeak analysis, ∼22 and ∼23% of the total events for K36-SfCherry-K36 and K36-SfGFP-K36, respectively, were observed with a distinguishable subpeak at the center, as shown in Figure 4d. This proportion is slightly lower than that of the protein-K72 series, which indicates that the portion of false positives caused by carrier folding was eliminated. These two samples have a mean subpeak amplitude of 35 ± 3 and 37 ± 3 pA, which are very similar to the ones measured for K72 carriers (Figure 4e). For both variants, the distribution of subpeak fractional position had a single peak at 0.5 as expected, corresponding to the protein (Figure 4f).

### Nanopore Sensing of Single Protein–Protein Interactions Using SUP Carriers

**Figure 5:**
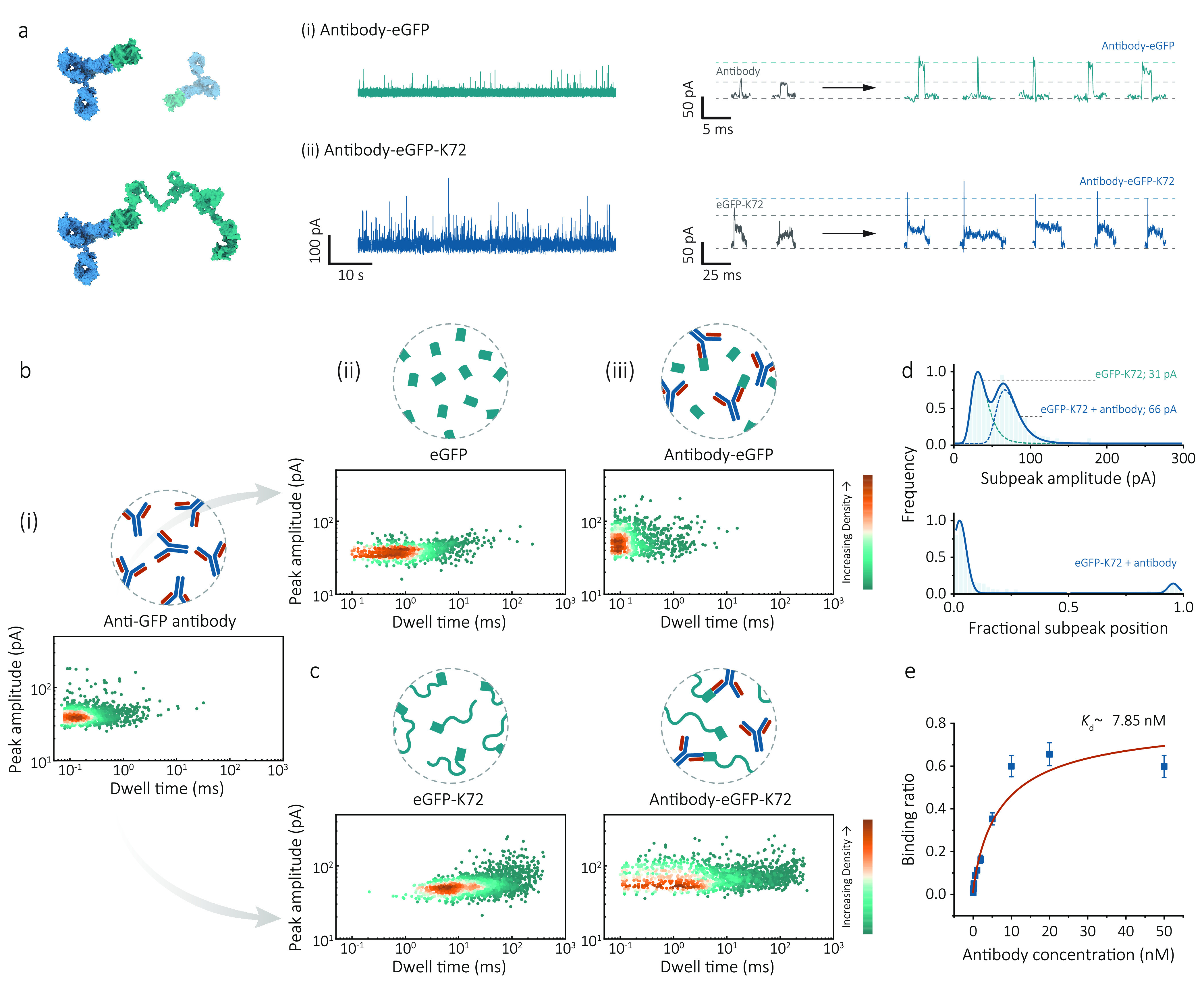
Nanopore sensing of single protein–protein interactions using cationic SUPs. (a) Proof-of-concept demonstrated here is using eGFP-K72 as the antigen to detect anti-GFP antibody. Schematic representation of antibody–eGFP complexes and antibody-eGFP-K72 complexes. Representative ionic current traces for (i) antibody (10 nM)-eGFP (1 nM) interactions and (ii) antibody (10 nM)-eGFP-K72 (1 nM) interactions recorded in PBS + 1 M KCl, 10 mM Tris-EDTA pH 8.0 buffer (1: 9, v/v) at −500 mV. Typical individual events show an enhanced subpeak for antibody-eGFP-K72 complexes compared with eGFP-K72. Data were sampled at 1 MHz and re-filtered to 5 kHz for visualization. (b) Schematic illustration of antibody-eGFP binding and density scatter plots of peak amplitude versus dwell time for separate translocation experiments of (i) 10 nM antibody (*n* = 1220), (ii) 750 nM eGFP (*n* = 845), and (iii) 10 nM antibody + 1 nM eGFP (*n* = 979). (c) Same antigen-antibody binding, but the antigen was replaced with eGFP-K72. Density scatter plots for separate experiments of 10 nM antibody (*n* = 1220), 1 nM eGFP-K72 (*n* = 1865), and 10 nM antibody + 1 nM eGFP-K72 (*n* = 1378). (d) Subpeak analysis of eGFP-K72 (1 nM) interacting with antibody (10 nM). Distribution of subpeak amplitude and fractional position (*n* = 425) showed the antibody bound with eGFP at the ends of SUP carriers resulting in a boost in subpeak amplitude. Threshold: subpeak amplitude >50 pA; dwell time >1 ms was selected to distinguish antibody-eGFP-K72 complexes from unbound states. (e) Binding assays of 1 nM eGFP-K72 in the presence of anti-GEP antibody ranging from 1 pM to 50 nM. The binding curve was fitted using the Hill equation, and the dissociation constant (*K*_d_ value) was determined to be ∼7.85 nM under this condition. Error bars represent one standard deviation of at least three independent experimental repeats.

Protein–protein interactions (PPIs) play an essential role in maintaining and modulating normal cellular processes, and thus, understanding these interactions provides valuable insights into the mechanism of protein-involved physiological processes and diseases.^49^ Recent advances in nanopore technology offer a promise of real-time recognition of PPIs at single-molecule resolution.^50^ However, inherently limited by the lack of sufficient spatiotemporal resolution, accurate identification of PPIs in direct nanopore measurements remains challenging. Here, compared with conventional nanopore measurements, the usage of cationic SUP carriers demonstrated improved detection of the translocated proteins. We replaced the original target protein with its protein-K72 form to obtain a higher capture efficiency and spatiotemporal resolution. In the presence of its interacting protein partner, we observed in an enhancement in the protein-bound subpeak due to the increase of analyte volume, which in turn confirms the occurrence of the desired PPI. For this purpose, we used the antigen–antibody interaction as a model. Specifically, eGFP-K72 was employed as the molecular probe for the recognition of anti-GFP antibody (IgG1, 146 kDa), while compared with the standard binding assay in which eGFP was bound to the antibody without the aid of SUP (K72) (Figure 5a–c).

To ensure efficient binding, eGFP and its antibody were incubated in a PBS buffer for two hours, and subsequently, resuspended in 1 M KCl Tris-EDTA buffer for nanopore measurements. Control translocation of anti-GFP antibody was first performed, as shown in Supporting Information, Figure S17. Even though the IgG antibody has a large molecular weight, its translocation speed was relatively quick, with a mean dwell time of 0.2 ± 0.1 ms. A well-defined single population with a mean value of 39 ± 2 pA was observed in the distribution of the peak amplitude of the antibody translocation. Despite the large molecular weight of the anti-GFP antibody, translocation of the antibody did not show a much deeper current blockade than the proteins measured previously. This anomaly is attributed to the nonglobular Y-shape.^51^ Addition of 1 nM eGFP with a stoichiometric ratio of 1:10 between the antigen and the antibody resulted in a wider peak amplitude distribution with an additional but not well-separated population at about 55 ± 3 pA (Supporting Information, Figure S18). However, it should be noted that these two populations showed similar dwell times that were very close to and potentially limited by the temporal resolution of the detection (at a low-pass filter of 10 kHz). Therefore, direct nanopore detection of the antigen–antibody complexes from a mixture remains a challenge (Figure 5b).

Since the SUP carrier is highly charged, nonspecific electrostatic interactions between the carrier and the target antibody might compete with the expected antigen–antibody interaction.^52^ Prior to investigating the interaction between eGFP-K72 and the antibody, K72 carrier was first incubated with the antibody, and almost no protein-bound-like subpeaks were observed in individual events, Supporting Information, Figure S19. Upon addition of excess antibody (10 nM) to a 1 nM eGFP-K72 solution, a part of the translocation events with a long dwell time were detected with an enhancement in the subpeak amplitude (Figure 5a and Supporting Information, Figure S20), which suggested the successful binding of antibody with eGFP-K72. In Figure 5c and Supporting Information, Figure S21, a comparison between the density scatter plots for the events attributed to eGFP-K72, antibody, antibody with eGFP, antibody with K72, and antibody with eGFP-K72. Unlike native eGFP bound with antibody, with the aid of K72 SUPs, the signals generated by the binding of eGFP-K72 with antibody could be well differentiated from free antibodies due to their two-level patterns. More details were presented in the distribution of the extracted subpeak amplitude, where two populations at 31 ± 3 and 66 ± 5 pA corresponded to the unbound eGFP and antibody-bound eGFP, respectively (Figure 5d). Hence, we could deduce a simple rule that the current blockade of the antigen–antibody complex approximately equals the sum of its separate peak value. Moreover, the enhancement of the subpeak amplitude only occurred, where the eGFP was present, confirming antibody–antigen specificity.

To determine the extent of antigen–antibody complex formation at different antigen–antibody ratios, a binding assay was performed as a function of antibody concentrations. In this case, the concentration of eGFP-K72 was kept at 1 nM, while the concentration of the target antibody was varied from 1 pM to 50 nM. The full binding curve fitted by the Hill–Langmuir equation is shown in Figure 5e, from which the dissociation constant (*K*_d_) was estimated to be 7.85 nM. The limit of detection (LOD), defined as three standard deviations (3σ) above background noise, was determined to be 74 pM calculated from the linear region within the low concentrations.

## Conclusions

We demonstrated that supercharged unstructured polypeptides can be used to sufficiently control protein transport through a nanopore. This peptide platform is genetically programmable, and hence, its charge density can be flexibly tuned depending on the practical purposes. In this work, both cationic and anionic SUPs can enhance the efficiency of protein capture due to the increase of the electrophoretic driving force within the local nanopore electric field. In particular, cationic SUPs enable an effective slowdown effect for the translocation of the protein attached, tackling a key limitation of spatiotemporal resolution in protein nanopore sensing. By investigating several cationic SUPs, it was confirmed that the nanopore transport slowdown is caused by electrostatic forces between the SUPs and the oppositely charged nanopore. Subpeak analysis allows to establish a correlation between the nanopore signals and the composition of target proteins, and the SUP carrier can be used to recognize protein–protein interactions at the single-molecule level. Furthermore, as the subpeak position corresponds well to the position of the protein attached, in principle, it may be possible to label multiple binding sites within a single SUP carrier for a high-throughput multiplexed detection. The improved capture efficiency may allow the analysis of rare proteins with low copy numbers during cellular processes, complementing the current advances in single-molecule proteomics.^15,16^ To extend our method to other proteins, it is planned to chemically attach modified SUPs to such targets using linker chemistry. Moreover, solid-state nanopores are easy to engineer, typically involving chemical modification of the nanopore surface^42,53^ or integrating the nanopore with other components, such as tunneling electrodes,^54,55^ field-effect transistors (FETs),^56,57^ and dielectrophoretic (DEP) traps,^37,54^ further advancing the performance. On the other hand, amino-acid-based recognition groups, such as antibodies, nanobodies, or peptide aptamers, can be easily fused with the SUP carrier by genetic encoding.

## Experimental Section

### Molecular Cloning

The gene monomers of SUPs were ordered from Integrated DNA Technologies (Iowa). One monomer corresponds to one building block of 9 charges. Sequences of genes and amino acid sequences are shown in Supporting Information Note 1. The gene fragments were ligated to the pJET1.2/blunt vector using T4 DNA ligase (Thermo Fisher Scientific) according to the blunt-end ligation protocol; the DNA fragment was used in a 3:1 molar ratio with the pJET1.2/blunt vector, and the ligation mixture was transformed after 1 h at 22 °C incubation. A **Van*91I* restriction site at the beginning and a **Bgl*I* restriction site at the end of the sequence were used for recursive directed ligation as described by Chilkoti et al.^33^ The recognition sites of the restriction enzymes **Van*91I* and **Bgl*I* were preserved by incorporating one valine instead of a charged amino acid residue per 10 pentapeptide repeats. The restriction was performed as described in the User Guide: Fast digestion of DNA (Thermo Fisher Scientific). One monomer plasmid was opened with **Van*91I* and alkaline phosphatase (FastAP; Thermo Fisher Scientific). A monomer of a second plasmid was cut out with **Van*91I* and **Bgl*I*. The resulting fragments were separated using a 1% agarose gel. The gel bands were cut out and purified using GFX PCR DNA and Gel Band Purification Kit (Cytiva). The restricted monomer fragment was ligated with T4 Ligase (Thermo Fisher Scientific) into the plasmid to form a dimer as described in the User Guide: Self-circularization of linear DNA. The plasmid was transformed into *E. coli* DH5α chemical competent cells and grown on LB agar plates with 100 μg/mL carbenicillin. Colonies were picked and grown in LB Lennox media overnight. Plasmid was extracted and isolated using the GeneJET Plasmid Miniprep kit. The DNA sequences were checked by sequencing (Microsynth Sequencing AG). The process was repeated up to an oligomerization of 8 building blocks. Therefore, 72 charges were located in the respective SUP. To clone the fusion proteins of eGFP and circularly permuted GFP with SUPs, the genes with the recognition sites of **Van*91I* and **Bgl*I* were ordered (IDT) and ligated to the pJET1.2/blunt vector as described before. The eGFP gene was cut with **Van*91I* and **Bgl*I* and cloned in a pJET SUP vector, which was opened with **Bgl*I*. Using the **Nde*I* and **Eco*RI* restriction site, the gene eGFP-SUP was cloned in the expression vector pET25b (+). In the case of pJET-CpGFP, the present **Bgl*I* site on the pJET vector was removed using the primers FW: 5′-CGC CGA GCG CAG AAG TGG TC-3′ and RV: 5′-CTG CCG GCT GGC TGG TTT ATT G-3′ because **Bgl*I* was used in further cloning. The ELP gene was cut by digestion with **Van*91I* and **Bgl*I* and run on a 1% agarose gel in TAE buffer. The band containing the ELP gene was excised from the gel and purified using a spin column purification kit (General Electric). pJET with target fragments were also digested with **Van*91I* and **Bgl*I* and dephosphorylated with FastAP. The vectors were purified by 1% agarose gel extraction. The linearized pJET vectors and the ELP-encoding gene were ligated using T4 ligase with a molar ratio 1:3 and transformed into chemically competent *E. coli* DH5α cells. Cells were plated and colonies were picked and grown in LB medium supplemented with 100 μg/mL carbenicillin overnight, and plasmids were isolated using the GeneJET Plasmid Miniprep kit. Positive clones were verified by analytical digest with **Van*91I* and **Bgl*I* following gel electrophoresis. The DNA sequences of the inserts were verified by DNA sequencing (Microsynth Sequencing AG). Gene oligomerization was again performed as described by Chilkoti and co-workers. Finally, the gene fragments encoding the ELP fusion proteins were transferred into the expression vector pET25b (+) for protein expression.

### Protein Expression and Purification

The *recA*-deficient variant *E. coli*. BLR (DE3) was used for protein expression. This strain has the ability to stabilize plasmids with repetitive sequences. To produce the target proteins, an LB overnight culture was diluted in TB medium (6% (w/v) tryptone, 12% (w/v) yeast extract, 85 mM KH_2_PO_4_, and 360 mM K_2_HPO_4_) to an OD600 of 0.1, supplemented with 100 μg/mL carbenicillin, and incubated in a shaking incubator at 37 °C and 200 rpm until OD600 0.6–0.8 was reached. The temperature was decreased to 30 °C and grown overnight. The cells had been centrifuged after harvesting at 4000*g*, 15 min, 4 °C, resuspended in lysis buffer (50 mM sodium phosphate buffer pH 7.0, 300 mM NaCl, 20 mM imidazole, EDTA-free protease inhibitor cocktail (cOmplete, Roche), 10 μg/mL DNaseI), and disrupted by a high pressure homogenizer (multi shot; Constant Systems Ltd). Insoluble cell debris were removed by centrifugation twice (14,000*g*, 1 h, 4 °C). The supernatant had been filtered through a pore size membrane filter (0.22 μm, Millipore), before target proteins were purified under native conditions by IMAC fast protein liquid chromatography (BioRad NGCTM) and loaded onto a pre-equilibrated (binding buffer: 50 mM sodium phosphate buffer pH 7.0, 300 mM NaCl, 20 mM Imidazole) Histrap fast flow column (5 mL, Cytiva). Subsequently, 5-column volumes were used to remove nonbinding impurities. Elution was carried out over 4 column volumes (CVs) with a gradient of 0 to 100% elution buffer (50 mM sodium phosphate buffer pH 7.0, 300 mM NaCl, 500 mM imidazole) and a flow rate of 1 mL/min. The fractions were collected with a volume of 1 mL. The product was further purified by ion exchange chromatography (*V* = 5 mL; Q HP column for glutamic acid-containing SUPs; heparin HP column for lysine-containing SUPs) on the already mentioned NGC chromatography system. The product fractions of previous IMAC chromatography were pooled and 10-fold diluted with ion exchange binding buffer (IEC A buffer: 50 mM phosphate buffer, 50 mM NaCl, pH = 7). The sample was loaded onto a pre-equilibrated ion exchange column. Unspecific binding proteins were washed out using 4 CV IEC A. The gradient elution proceeded from 0–100% elution buffer (50 mM phosphate buffer, 2 M NaCl, pH = 7) within 4 CV with a flow rate of 1.0 mL/min. Protein purity was determined on 12% SDS-PAGE stained with Coomassie staining solution (30% ethanol, 20% glacial acetic acid, 0.05% Brilliant Blue G250, 0.05% Brilliant Blue R250) and destained with destaining solution (40% EtOH, 10% acetic acid).

### Fabrication of Nanopipettes

Quartz capillaries (GQF100-50-7.5, World Precision Instruments, U.K.) with an outer diameter of 1.0 mm and an inner diameter of 0.5 mm with an inner filament were plasma-cleaned (Harrick Plasma) and then pulled by a laser-based pipette puller (Sutter Instrument, P-2000). Nanopipettes used in all nanopore experiments were fabricated through a two-line protocol: (1) HEAT = 825, FIL = 4, VEL = 30, DEL = 130, PUL = 80 and (2) HEAT = 850, FIL = 3, VEL = 20, DEL = 127, PUL = 185. It should be noted that these parameters are instrumentally specific and were optimized to yield nanopore openings of 12 ± 2 nm.

### Nanopore Measurements and Data Processing

The buffer used in the translocation experiments consisted of 1 M KCl and 10 mM Tris-EDTA (pH = 8), unless noted otherwise. For the binding assays, 1 nM eGFP-K72 was used and incubated with anti-GFP antibody (Thermo Fisher Scientific) at different concentrations for at least 2 h prior to nanopore experiments. Approximately 10 μL of the electrolyte was filed inside the nanopipette via a MicroFil needle (MF34G, World Precision Instruments, U.K.). Freshly made Ag/AgCl electrodes were then inserted into the nanopipette (trans chamber) and the bath (cis chamber), respectively. All ionic current recordings were performed using a high-bandwidth amplifier VC100 (Chimera Instruments). The recorded data were resampled to 1 MHz and low-pass filtered at 10 kHz. Analysis of all translocation events was performed using a custom-written MATLAB code, namely, The Nanopore App, credited to Professor J.B.E. A workflow of the analysis procedure is shown in Supporting Information Figure S22. Subpeak amplitudes extracted from the events with a two-level signature were defined as the maximum peak current minus the SUP level.

## Supporting Information Available

Sequences of SUPs and protein–SUP complexes, preparation and characterization of SUP samples; characterization of nanopipettes; additional nanopore analysis of SUPs; identification of protein–protein interactions with SUP and without SUP; and workflow of data analysis (PDF)
