# Supplementary material for: Nanopore Detection Using Supercharged Polypeptide Molecular Carriers
Source: J Am Chem Soc. 2023 Mar 10;145(11):6371–82. doi: 10.1021/jacs.2c13465 (PMC10037339; doi:10.1021/jacs.2c13465)
Supplement: Supplementary file 1 — ja2c13465_si_001.pdf [file ja2c13465_si_001.pdf]

Supporting Information

Nanopore detection using supercharged polypeptide molecular carriers

Xiaoyi Wang [a‡], Tina-Marie Thomas [b, c‡], Ren Ren [a, f], Yu Zhou [b, c], Peng Zhang [d], Jingjing Li [d], Shenglin Cai [a], Kai Liu [e], Aleksandar P. Ivanov *[a], Andreas Herrmann *[b, c], Joshua B. Edel *[a]

a Department of Chemistry, Imperial College London, Molecular Science Research Hub, London, W12 0BZ, UK.

b DWI – Leibniz Institute for Interactive Materials, Forckenbeckstr. 50, 52056 Aachen, Germany.

c Institute of Technical and Macromolecular Chemistry, RWTH Aachen University, Worringerweg 1, 52074 Aachen, Germany.

d State Key Laboratory of Rare Earth Resource Utilization, Changchun Institute of Applied Chemistry, Chinese Academy of Sciences, Changchun, 130022, China.

e Engineering Research Center of Advanced Rare Earth Materials, (Ministry of Education), Department of Chemistry, Tsinghua University, Beijing, 100084, China.

f Department of Metabolism, Digestion and Reproduction, Imperial College London, London, W12 0NN, UK.

‡These authors contributed equally.

Table of contents

Section S1. Preparation and characterization of SUPs and protein-SUP complexes

Section S2. Characterization of nanopipettes

Section S3. Nanopore translocation of protein-SUPs

Section S4. Quantification of single protein-protein interactions using protein-SUP probes

Section S5. Data analysis

Section S1. Preparation and characterization of SUPs and protein-SUP complexes

Supplementary Table 1. SUP samples used in this work

Name	Isoelectric point (pI)	Sequence	Molecular weight (kDa)
eGFP	6.0	eGFP	27.4
eGFP-E36	4.57	eGFP-$(GEGVP)_{36}H_6$	46.6
eGFP-K18	9.8	eGFP-$(GKGVP)_{18}H_6$	37.9
eGFP-K36	10.3	eGFP-$(GKGVP)_{36}H_6$	46.6
GFP-K72	10.7	eGFP-$(GKGVP)_{72}H_6$	64.0
K72	11.85	$(GKGVP)_{72}H_6$	36.4
V40K72	11.85	$(GVGVP)_{40}(GKGVP)_{72}H_6$	52.8
CpsfCherry2-K72	10.15	CpsfCherry2-$(GKGVP)_{72}H_6$	62.9
Sn-K72	10.98	Sn-$(GKGVP)_{72}H_6$	44.1
mIFP-K72	10.45	mIFP-$(GKGVP)_{72}H_6$	73.0
K36-SfGFP-K36	10.7	$(GKGVP)_{36}$-SfGFP-$(GKGVP)_{36}H_6$	63.8
K36-SfCherry2-K36(K198C)	10.17	$(GKGVP)_{36}$- SfCherry2-$(GKGVP)_{36}H_6$	62.5

Supplementary Note 1. Sequences of SUPs and protein-SUP complexes

eGFP

MASKGEELFTGVVPILVELDGDVNGHKFSVSGEGEGDATYGKLTLKFICTTGKLPVPWPTLVTTLTYGVQCFSRYPDHMKRHDFFKSAMPE
GYVQERTISFKDDGNYKTRAEVKFEGDTLVNRIELKGIDFKEDGNILGHKLEYNYNSHNVYITADKQKNGIKANFKIRHNIEDGSVQLADHYQ
QNTPIGDGPVLLPDNHYLSTQSALSKDPNEKRDHMVLLEFVTAAGITHGMDELHHHHHH

Size: 243 AAs

MW: 27378.77 Da

pI: 6.0

Net charge: -6.6

eGFP-E36

MASKGEELFTGVVPILVELDGDVNGHKFSVSGEGEGDATYGKLTLKFICTTGKLPVPWPTLVTTLTYGVQCFSRYPDHMKRHDFFKSAMPE
GYVQERTISFKDDGNYKTRAEVKFEGDTLVNRIELKGIDFKEDGNILGHKLEYNYNSHNVYITADKQKNGIKANFKIRHNIEDGSVQLADHYQ
QNTPIGDGPVLLPDNHYLSTQSALSKDPNEKRDHMVLLEFVTAAGITHGMDELGVVGLVPRGSHMGAGPGVGVPGEGVPGEGVPGEGV
PGEGVPGEGVPGEGVPGEGVPGEGVPGEGVPGVGVPGEGVPGEGVPGEGVPGEGVPGEGVPGEGVPGEGVPGEGVPGEGVPGVGVP
GEGVPGEGVPGEGVPGEGVPGEGVPGEGVPGEGVPGEGVPGEGVPGVGVPGEGVPGEGVPGEGVPGEGVPGEGVPGEGVPGEGVPG
EGVPGEGVPGWPHHHHHH

Size: 462 AAs

MW: 46650.65 Da

pI: 4.57

Net charge: -41.5

eGFP-K18

MASKGEELFTGVVPILVELDGDVNGHKFSVSGEGEGDATYGKLTLKFICTTGKLPVPWPTLVTTLTYGVQCFSRYPDHMKRHDFFKSAMPE
GYVQERTISFKDDGNYKTRAEVKFEGDTLVNRIELKGIDFKEDGNILGHKLEYNYNSHNVYITADKQKNGIKANFKIRHNIEDGSVQLADHYQ
QNTPIGDGPVLLPDNHYLSTQSALSKDPNEKRDHMVLLEFVTAAGITHGMDELGVVGLVPRGSHMGAGPGVGVPGKGVPGKGVPGKGV
PGKGVPGKGVPGKGVPGKGVPGKGVPGKGVPGVGVPGKGVPGKGVPGKGVPGKGVPGKGVPGKGVPGKGVPGKGVPGKGVPGWPH
HHHHH

Size: 362 AAs

MW: 37904.29 Da

pI: 9.8

Net charge: +12.5

eGFP-K36

MASKGEELFTGVVPILVELDGDVNGHKFSVSGEGEGDATYGKLTLKFICTTGKLPVPWPTLVTTLTYGVQCFSRYPDHMKRHDFFKSAMPE
GYVQERTISFKDDGNYKTRAEVKFEGDTLVNRIELKGIDFKEDGNILGHKLEYNYNSHNVYITADKQKNGIKANFKIRHNIEDGSVQLADHYQ
QNTPIGDGPVLLPDNHYLSTQSALSKDPNEKRDHMVLLEFVTAAGITHGMDELGVVGLVPRGSHMGAGPGVGVPGKGVPGKGVPGKGV
PGKGVPGKGVPGKGVPGKGVPGKGVPGKGVPGVGVPGKGVPGKGVPGKGVPGKGVPGKGVPGKGVPGKGVPGKGVPGKGVPGVGVP
GKGVPGKGVPGKGVPGKGVPGKGVPGKGVPGKGVPGKGVPGKGVPGVGVPGKGVPGKGVPGKGVPGKGVPGKGVPGKGVPGKGVPG
KGVPGKGVPGWPHHHHHH

Size: 462 AAs

MW: 46616.5 Da

pI: 10.3

Net charge: +30.5

eGFP-K72

MASKGEELFTGVVPILVELDGDVNGHKFSVSGEGEGDATYGKLTLKFICTTGKLPVPWPTLVTTLTYGVQCFSRYPDHMKRHDFFKSAMPE
GYVQERTISFKDDGNYKTRAEVKFEGDTLVNRIELKGIDFKEDGNILGHKLEYNYNSHNVYITADKQKNGIKANFKIRHNIEDGSVQLADHYQ
QNTPIGDGPVLLPDNHYLSTQSALSKDPNEKRDHMVLLEFVTAAGITHGMDELGVVGLVPRGSHMGAGPGVGVPGKGVPGKGVPGKGV
PGKGVPGKGVPGKGVPGKGVPGKGVPGKGVPGVGVPGKGVPGKGVPGKGVPGKGVPGKGVPGKGVPGKGVPGKGVPGKGVPGVGVP
GKGVPGKGVPGKGVPGKGVPGKGVPGKGVPGKGVPGKGVPGKGVPGVGVPGKGVPGKGVPGKGVPGKGVPGKGVPGKGVPGKGVPG
KGVPGKGVPGVGVPGKGVPGKGVPGKGVPGKGVPGKGVPGKGVPGKGVPGKGVPGKGVPGVGVPGKGVPGKGVPGKGVPGKGVPGK
GVPGKGVPGKGVPGKGVPGKGVPGVGVPGKGVPGKGVPGKGVPGKGVPGKGVPGKGVPGKGVPGKGVPGKGVPGVGVPGKGVPGKG
VPGKGVPGKGVPGKGVPGKGVPGKGVPGKGVPGKGVPGWPHHHHHH

Size: 664 AAs

MW: 64041.6 Da

pI: 10.7

Net charge: +66.5

K72

MGAGPGVGVPGKGVPGKGVPGKGVPGKGVPGKGVPGKGVPGKGVPGKGVPGKGVPGVGVPGKGVPGKGVPGKGVPGKGVPGKGVP
GKGVPGKGVPGKGVPGKGVPGVGVPGKGVPGKGVPGKGVPGKGVPGKGVPGKGVPGKGVPGKGVPGKGVPGVGVPGKGVPGKGVPG
KGVPGKGVPGKGVPGKGVPGKGVPGKGVPGKGVPGVGVPGKGVPGKGVPGKGVPGKGVPGKGVPGKGVPGKGVPGKGVPGKGVPGV
GVPGKGVPGKGVPGKGVPGKGVPGKGVPGKGVPGKGVPGKGVPGKGVPGVGVPGKGVPGKGVPGKGVPGKGVPGKGVPGKGVPGKG
VPGKGVPGKGVPGVGVPGKGVPGKGVPGKGVPGKGVPGKGVPGKGVPGKGVPGKGVPGKGVPGWPHHHHHH

Size: 414 AAs

MW: 36444.58 Da

pI: 11.85

Net charge: +72

V40K72

MGAGPGVGVPGVGVPGVGVPGVGVPGVGVPGVGVPGVGVPGVGVPGVGVPGVGVPGVGVPGVGVPGVGVPGVGVPGVGVPGVGVPGVGVPGVGVPGVGV
PGVGVPGVGVPGVGVPGVGVPGVGVPGVGVPGVGVPGVGVPGVGVPGVGVPGVGVPGVGVPGVGVPGVGVPGVGVPGVGVPGVGVPGVGVPGVGVPGVG
VPGVGVPGVGVPGVGVPGVGVPGVGVPGVGVPGVGVPGVGVPGVGVPGKGVPGKGVPGKGVPGKGVPGKGVPGKGVPGKGVPGKGVPGKG
VPGVGVPGKGVPGKGVPGKGVPGKGVPGKGVPGKGVPGKGVPGKGVPGKGVPGVGVPGKGVPGKGVPGKGVPGKGVPGKGVPGKGV
PGKGVPGKGVPGKGVPGVGVPGKGVPGKGVPGKGVPGKGVPGKGVPGKGVPGKGVPGKGVPGKGVPGVGVPGKGVPGKGVPGKGVP
GKGVPGKGVPGKGVPGKGVPGKGVPGKGVPGVGVPGKGVPGKGVPGKGVPGKGVPGKGVPGKGVPGKGVPGKGVPGKGVPGVGVPG
KGVPGKGVPGKGVPGKGVPGKGVPGKGVPGKGVPGKGVPGKGVPGVGVPGKGVPGKGVPGKGVPGKGVPGKGVPGKGVPGKGVPGK
GVPGKGVPGWPHHHHHH

Size: 614 AAs

MW: 52824.91 Da

pI: 11.85

Net charge: +72

CpsfCherry2-K72(K198C)

MHHHHHHGGGSYTIVEQYERAEARHSTGGGSGGGSHGVGVPGKGVPGKGVPGKGVPGKGVPGKGVPGKGVPGKGVPGKGVPGKGVP
GVGVPGKGVPGKGVPGKGVPGKGVPGKGVPGKGVPGKGVPGKGVPGKGVPGVGVPGKGVPGKGVPGKGVPGKGVPGKGVPGKGVPG
KGVPGKGVPGKGVPGVGVPGKGVPGKGVPGKGVPGKGVPGKGVPGKGVPGKGVPGKGVPGKGVPGVGVPGKGVPGKGVPGKGVPGK
GVPGKGVPGKGVPGKGVPGKGVPGKGVPGVGVPGKGVPGKGVPGKGVPGKGVPGKGVPGKGVPGKGVPGKGVPGKGVPGVGVPGKG
VPGKGVPGKGVPGKGVPGKGVPGKGVPGKGVPGKGVPGKGVPGVGVPGKGVPGKGVPGKGVPGKGVPGKGVPGKGVPGKGVPGKGV
PGKGVPGRPGGGSGGGSMEEDNMAIIKEFMRFKVHMEGSVNGHEFEIEGEGEGHPYEGTQTAKLKVTKGGPLPFAWDILSPQFMYGSK
AYVKHPADIPDYLKLSFPEGFTWERVMNFEDGGVVTVTQDSSLQDGQFIYKVKLLGINFPSDGPVMQKKTMGWEASTERMYPEDGALKG
EINQRLKLKDGGHYDAEVKTTYKAKKPVQLPGAYNVDICLDITSHNED

Size: 655 AAs

MW: 62911.15 Da

pI: 10.15

Net charge: +63.2

Sn-K72

MAGGCKGADGAHGVNGCPGTAGAAGSVGGPGCDGGHGGNGGNGNPGCAGGVGGAGGASGGTGVGGRGGKGGSGTPKGADGAP
GAPAGGGGSGGGGSHMGAGPGVGVPGKGVPGKGVPGKGVPGKGVPGKGVPGKGVPGKGVPGKGVPGKGVPGVGVPGKGVPGKGV
PGKGVPGKGVPGKGVPGKGVPGKGVPGKGVPGKGVPGVGVPGKGVPGKGVPGKGVPGKGVPGKGVPGKGVPGKGVPGKGVPGKGVP
GVGVPGKGVPGKGVPGKGVPGKGVPGKGVPGKGVPGKGVPGKGVPGKGVPGKGVPGVGVPGKGVPGKGVPGKGVPGKGVPGKGVPGKGVPG
KGVPGKGVPGKGVPGVGVPGKGVPGKGVPGKGVPGKGVPGKGVPGKGVPGKGVPGKGVPGKGVPGVGVPGKGVPGKGVPGKGVPGK
GVPGKGVPGKGVPGKGVPGKGVPGKGVPGVGVPGKGVPGKGVPGKGVPGKGVPGKGVPGKGVPGKGVPGKGVPGKGVPGWPHHHH
HH

Size: 511 AAs

MW: 44065.54 Da

pI: 10.98

mIFP-K72

MASSVPLTTSAFGHAFLANCEREQIHLAGSIQPHGILLAVKEPDNVVIQASINAAEFLNTNSVVGRPLRDLGGDLPLQILPHLNGPLHLAPMT
LRCTVGSPPRRVDCTIHRPSNGGLIVELEPATKTTNIAPALDGAFHRITSSSSLMGLCDETATIIREITGYDRVMVVRFDEEGNGEILSERRRAD
LEAFLGNRYPASTIPQIARLLYEHNRVRLLVDVNYTPVPLQPRISPLNGRDLDMSLSCLRSMSPIHQKYMQDMGVGATLVCSLMVSGRLWG
LIACHHYEPRFVPFHIRAAGEALAETCAIRIATLESFAQSQSKELGVVGLVPRGSHMGAGPGVGVPGKGVPGKGVPGKGVPGKGVPGKGVP
GKGVPGKGVPGKGVPGKGVPGVGVPGKGVPGKGVPGKGVPGKGVPGKGVPGKGVPGKGVPGKGVPGKGVPGVGVPGKGVPGKGVPG
KGVPGKGVPGKGVPGKGVPGKGVPGKGVPGKGVPGVGVPGKGVPGKGVPGKGVPGKGVPGKGVPGKGVPGKGVPGKGVPGKGVPGV
GVPGKGVPGKGVPGKGVPGKGVPGKGVPGKGVPGKGVPGKGVPGKGVPGVGVPGKGVPGKGVPGKGVPGKGVPGKGVPGKGVPGKG
VPGKGVPGKGVPGVGVPGKGVPGKGVPGKGVPGKGVPGKGVPGKGVPGKGVPGKGVPGKGVPGVGVPGKGVPGKGVPGKGVPGKGV
PGKGVPGKGVPGKGVPGKGVPGKGVPGWPHHHHHH

Size: 749 AAs

MW: 72953.66 Da

pI: 10.45

K36-SfGFP-K36

HMSHGVGVPGKGVPGKGVPGKGVPGKGVPGKGVPGKGVPGKGVPGKGVPGVGVPGKGVPGKGVPGKGVPGKGVPGKGVPG
KGVPGKGVPGKGVPGKGVPGVGVPGKGVPGKGVPGKGVPGKGVPGKGVPGKGVPGKGVPGKGVPGKGVPGVGVPGKGVPGKGVPGK
GVPGKGVPGKGVPGKGVPGKGVPGKGVPGKGVPGKGVPGVGGGGSGGGSMSKGEELFTGVVPILVELDGDVNGHKFSVRGEGEGDATIGKLTL
KFIATTGKLPVPWPTLVTTFSYGVQCFSRYPDHMKQHDFFKSAMPEGYVQERTIFFKDDGKYKTRAVVKFEGDTLVNRIELKGTDFKEDGNI
LGHKLEYNFNSHNVYIMADKQKNGIKVNFTVRHNVEDGSVQLADHYQQNTPIGDGPVLLPDNHYLSTCTVLSKDPNEKRDHMVLHEYVN
AAGITHGMDELYKGGGSGGGSWPGVPGKGVPGKGVPGKGVPGKGVPGKGVPGKGVPGKGVPGKGVPGKGVPGVGVPGKGVPGKGVP
GKGVPGKGVPGKGVPGKGVPGKGVPGKGVPGKGVPGVGVPGKGVPGKGVPGKGVPGKGVPGKGVPGKGVPGKGVPGKGVPGKGVPG
VGVPGKGVPGKGVPGKGVPGKGVPGKGVPGKGVPGKGVPGKGVPGKGVPGKGVPGVG

Size: 664 AAs

MW: 63773.28 Da

pI: 10.7

Net charge: +67.1

K36-SfCherry2-K36(K198C)

HMSHGVGVPGKGVPGKGVPGKGVPGKGVPGKGVPGKGVPGKGVPGKGVPGVGVPGKGVPGKGVPGKGVPGKGVPGKGVPG
KGVPGKGVPGKGVPGKGVPGVGVPGKGVPGKGVPGKGVPGKGVPGKGVPGKGVPGKGVPGKGVPGKGVPGVGVPGKGVPGKGVPGK
GVPGKGVPGKGVPGKGVPGKGVPGKGVPGKGVPGKGVPGRPGGGSGGGSMEEDNMAIIKEFMRFKVHMEGSVNGHEFEIEGEGEGHPYEGTQ
TAKLKVTKGGPLPFAWDILSPQFMYGSKAYVKHPADIPDYLKLSFPEGFTWERVMNFEDGGVVTVTQDSSLQDGQFIYKVKLLGINFPSDGP
VMQKKTMGWEASTERMYPEDGALKGEINQRLKLKDGGHYDAEVKTTYKAKKPVQLPGAYNVDICLDITSHNEDYTIVEQYERAEARHSTG
GGSGGGSWPGVPGKGVPGKGVPGKGVPGKGVPGKGVPGKGVPGKGVPGKGVPGKGVPGVGVPGKGVPGKGVPGKGVPGKGVPGKG
VPGKGVPGKGVPGKGVPGKGVPGVGVPGKGVPGKGVPGKGVPGKGVPGKGVPGKGVPGKGVPGKGVPGKGVPGVGVPGKGVPGKGV
PGKGVPGKGVPGKGVPGKGVPGKGVPGKGVPGKGVPGRP

Size: 650 AAs

MW: 62491.79 Da

pI: 10.17

Net charge: +63.8

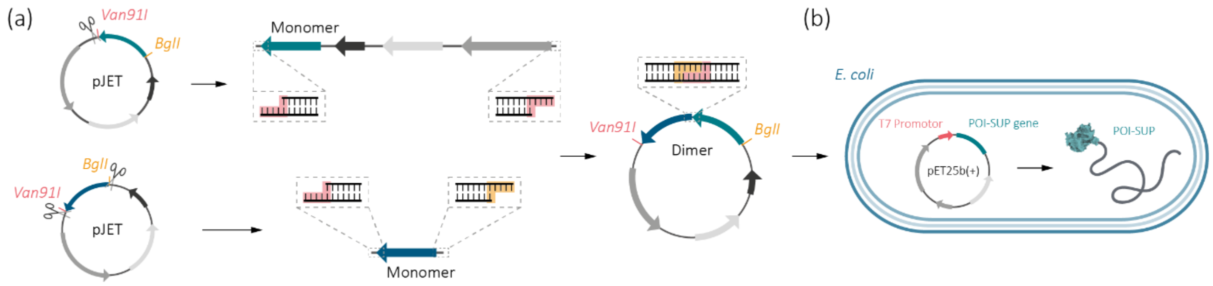

Figure S1. Schematic for the preparation of SUPs via recombinant DNA technology. (a) Recursive directional ligation (RDL) was used for the molecular cloning of SUP plasmid. (b) The fused SUPs were expressed in *E. coli* system.

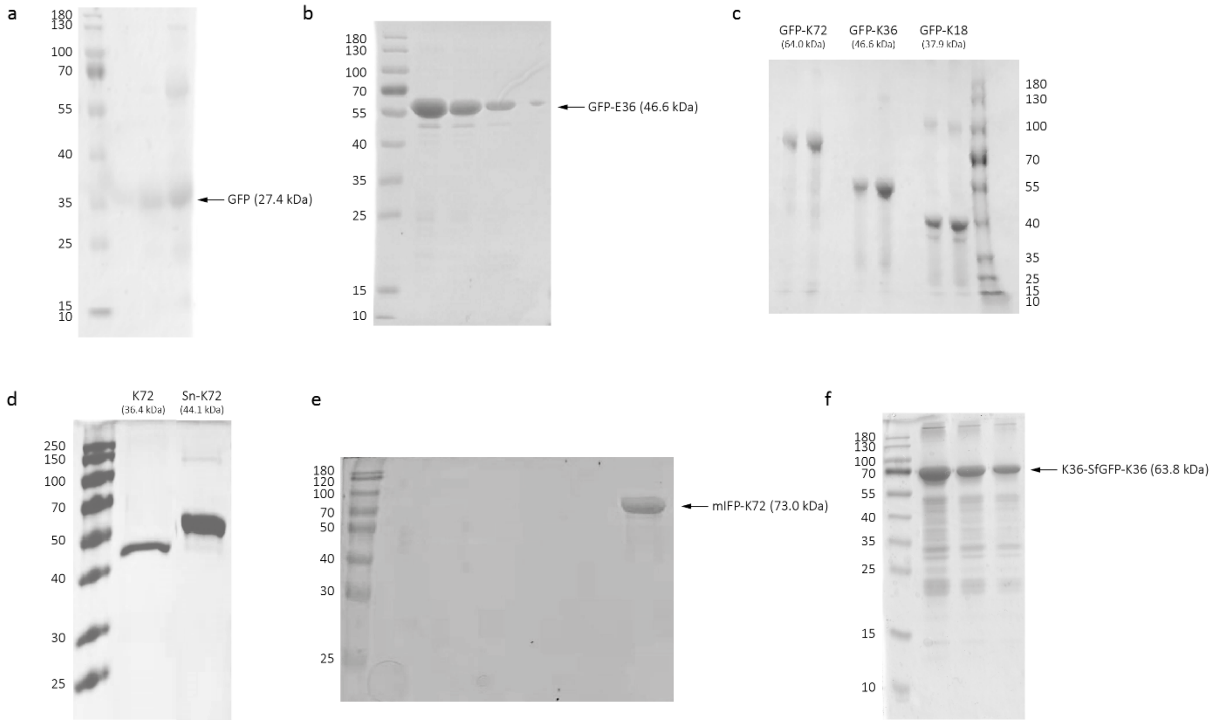

Figure S2. SDS-PAGE characterization of SUP samples. Protein Ladder molecular weight markers were shown in each gel image. (a) eGFP; (b) eGFP-E36; (c) eGFP-K18, eGFP-K36, and eGFP-K72; (d) K72 and Sn-K72; (e) mIFP-K72; (f) K36-SfGFP-K36. It should be noted that the electrophoretic mobility of highly charged SUP samples are slightly different from folded proteins, which usually exhibit a larger apparent mass than standard mass markers.

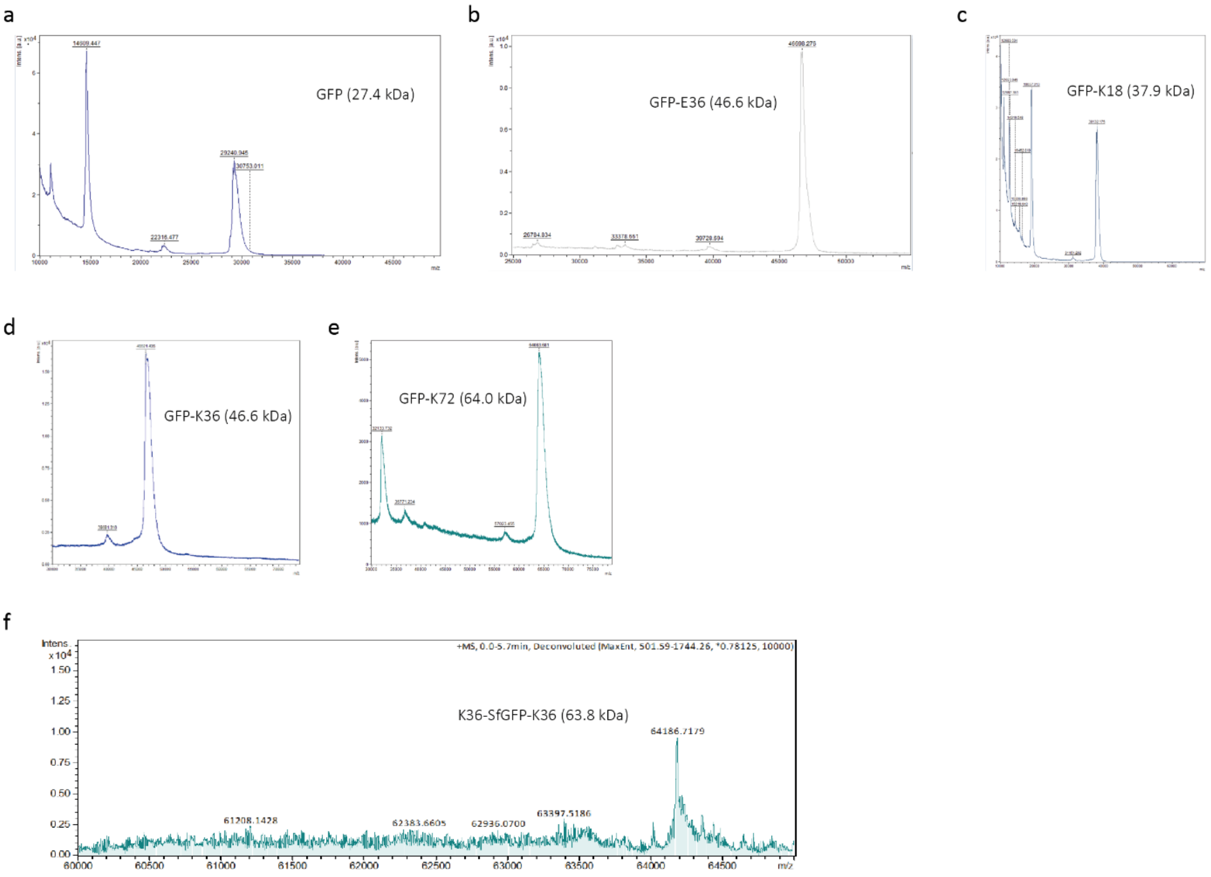

Figure S3. MALDI-TOF mass spectra of SUP samples. (a) eGFP; (b) eGFP-E36; (c) eGFP-K18; (d) eGFP-K36; (e) eGFP-K72; (f) K36-SfGFP-K36.

Section S2. Characterization of nanopipettes

Supplementary Note 2. Approximation of the diameter of nanopipettes

Nanopipettes were approximated as truncated hollow cones in order to model their resistive properties. The resistance then was determined by its inner cone angle and aperture radius according to the equation:[1]

$$R_\mathrm{p} = \frac{1}{\kappa \pi r_\mathrm{i} \tan\theta} + R_\mathrm{access} \approx \frac{1}{\kappa \pi r_\mathrm{i} \tan\theta} + \frac{1}{4\kappa r_\mathrm{i}}$$

where R_p is the nanopipette resistance, r_i is the inner pipette radius, κ is the solution conductivity, and θ is the inner nanopipette half-cone angle.

In this work, the nanopipette resistance was calculated by fitting the *I-V* data, shown in Figure S4. The inner half cone angle of nanopipettes was measured using SEM, imaging the top 100 nm of the pipette tip.

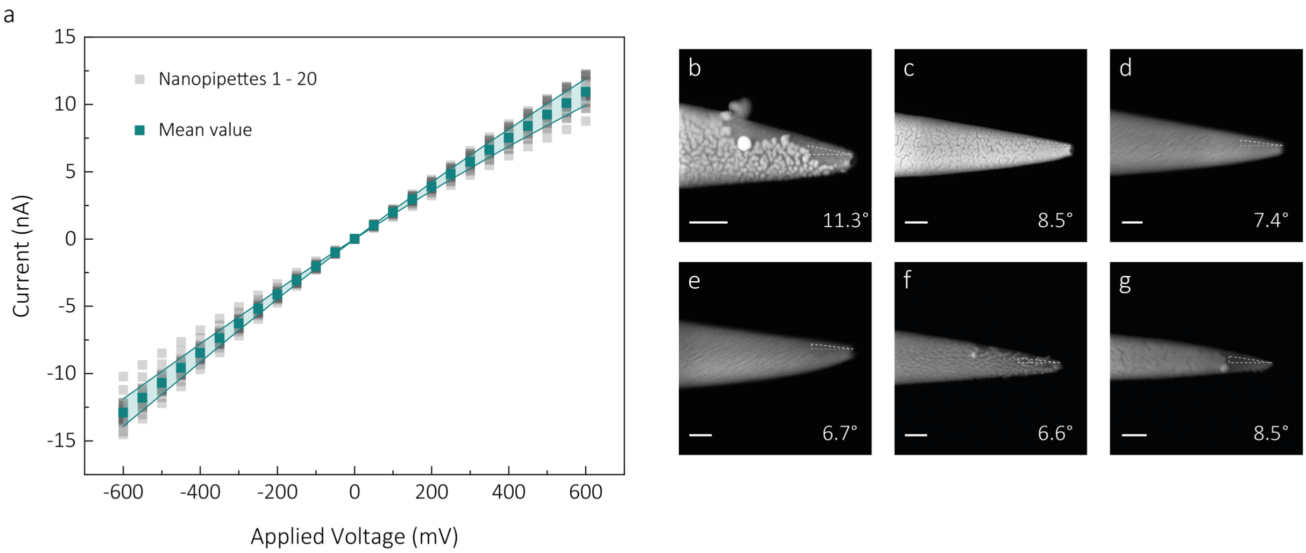

Figure S4. Conductance characterization of nanopipettes. The nanopipettes used in this work were fabricated from quartz capillaries (ID: 0.5 mm, OD: 1.0 mm) by a laser-assisted puller. (a) I-V curves were measured in 1 M KCl, 10 mM Tris-EDTA, pH 8.0 buffer. The nanopore conductance was estimated to be 20.0 ± 1.6 nS fitted as a linear curve. (b-g) SEM images of the nanopipettes show the taper and geometry (scale bar: 100 nm). The outer half cone angle were directly measured from these side-view images. We assumed the inner semi-angle is equal to the outer semi-angle when calculating the nanopore size.

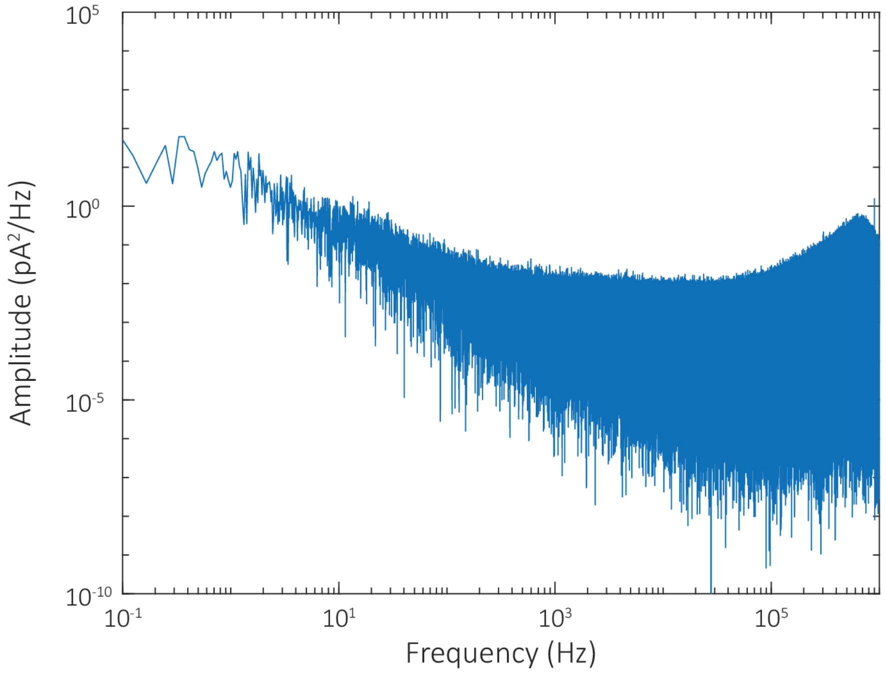

Figure S5. Power spectral density (PSD) plots of the nanopore platform. PSD plots were measured in 1 M KCl, 10 mM Tris-EDTA, pH 8.0 buffer, under a voltage of 500 mV.

Section S3. Nanopore translocation of protein-SUPs

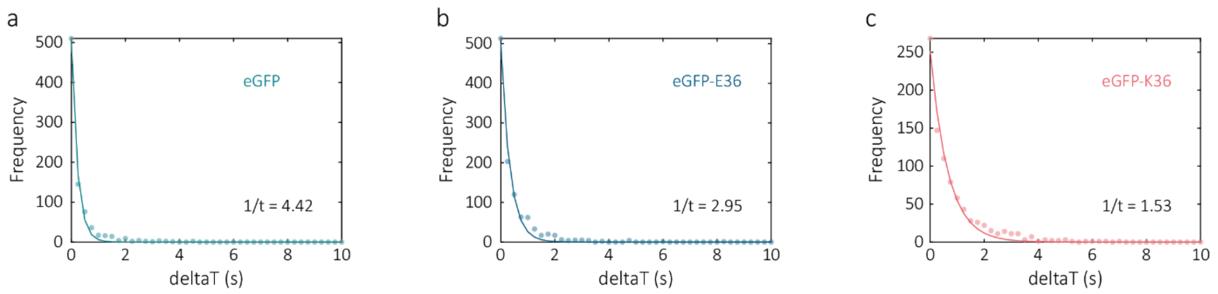

Figure S6. Capture rate of eGFP, eGFP-E36 and eGFP-K36. Distributions of the inter-event time (the time between successive captured events) for (a) 750 nM eGFP, (b) 5 nM eGFP-E36, and (c) 5 nM eGFP-K36 at an applied voltage of 500 mV in 1 M KCl, 10 mM Tris-EDTA, pH 8.0 buffer. Solid lines represent single-exponential fits, from which the protein capture rate was extracted.

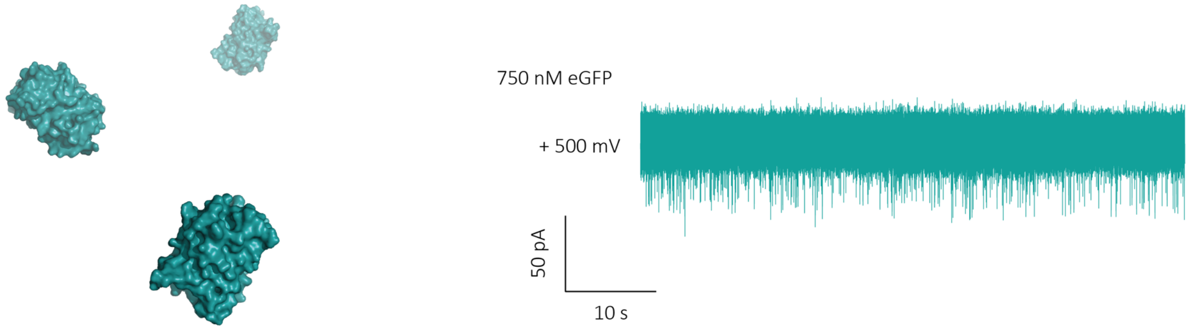

Figure S7. eGFP nanopore detection at a high concentration. 750 nM eGFP was used to obtain sufficient data for statistics due to the low capture rate for native eGFP. Protein translocation was performed in 1 M KCl, 10 mM Tris-EDTA, pH 8.0 buffer at +500 mV.

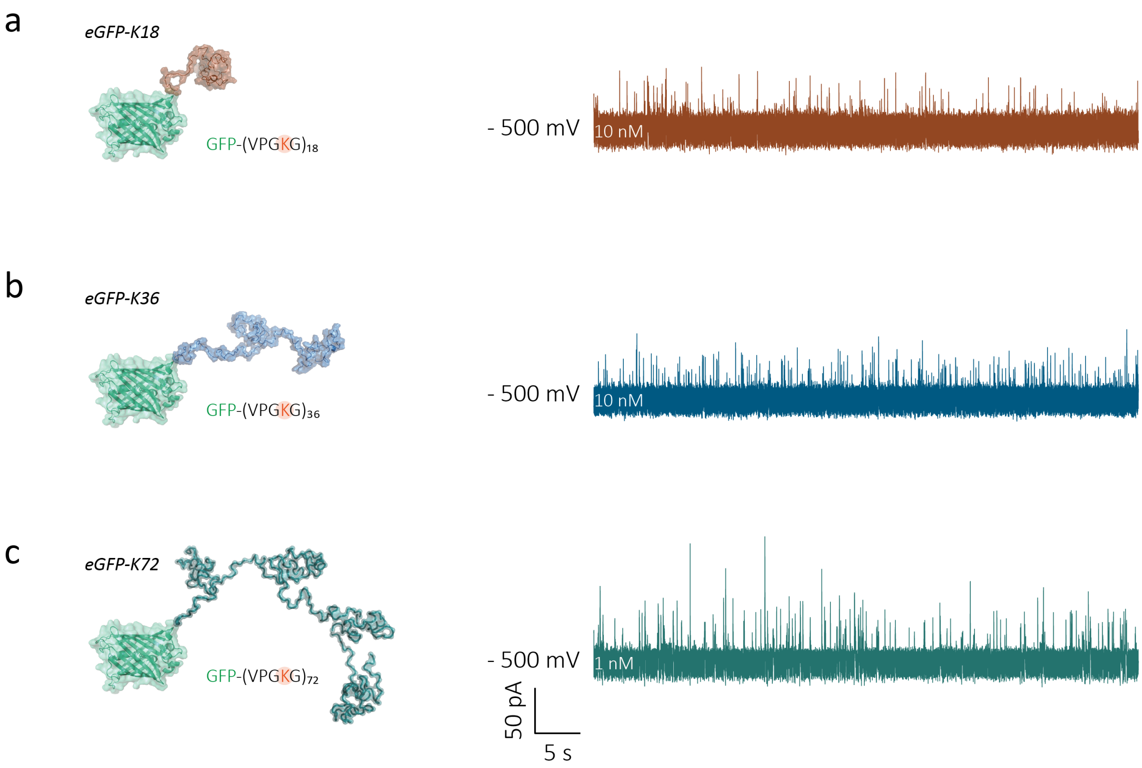

Figure S8. Nanopore detection of eGFP-K18, eGFP-K36 and eGFP-K72. Representative traces for the translocation of (a) 10 nM eGFP-K18, (b) 10 nM eGFP-K36, and (c) 1 nM eGFP-K72. All the translocation experiments were performed in 1 M KCl, 10 mM Tris-EDTA, pH 8.0 buffer at -500 mV. Data were sampled at 1 MHz and digitally filtered using a Bessel low-pass filter at 10 kHz.

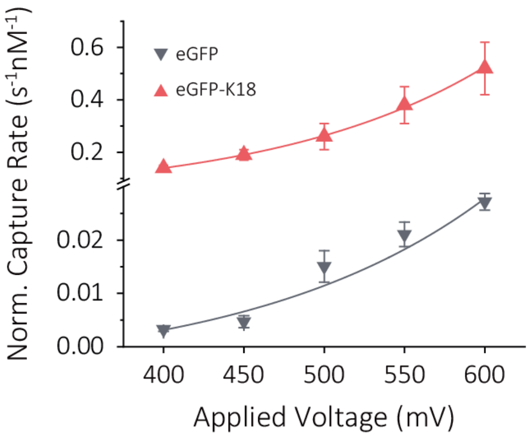

Figure S9. Voltage dependence of capture rate for eGFP and eGFP-K18. Normalized capture rate of cationic eGFP-SUPs was plotted as a function of the applied voltage. In particular, eGFP and eGFP-K18 show an exponential increase with the voltage, which indicates its nanopore translocation is limited by an energy barrier. Longer molecules, eGFP-K36 and eGFP-K72, present a linear increase with voltage.

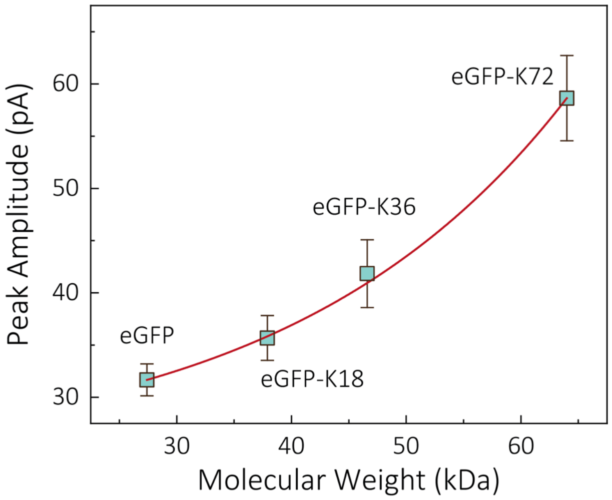

Figure S10. Dependence of peak amplitude on molecular weight. Peak amplitude of eGFP variants as a function of molecular weight. The current amplitude shows an exponential increase in response to the increasing length of SUPs. This experiment was repeated at least three times for each protein at 500 mV.

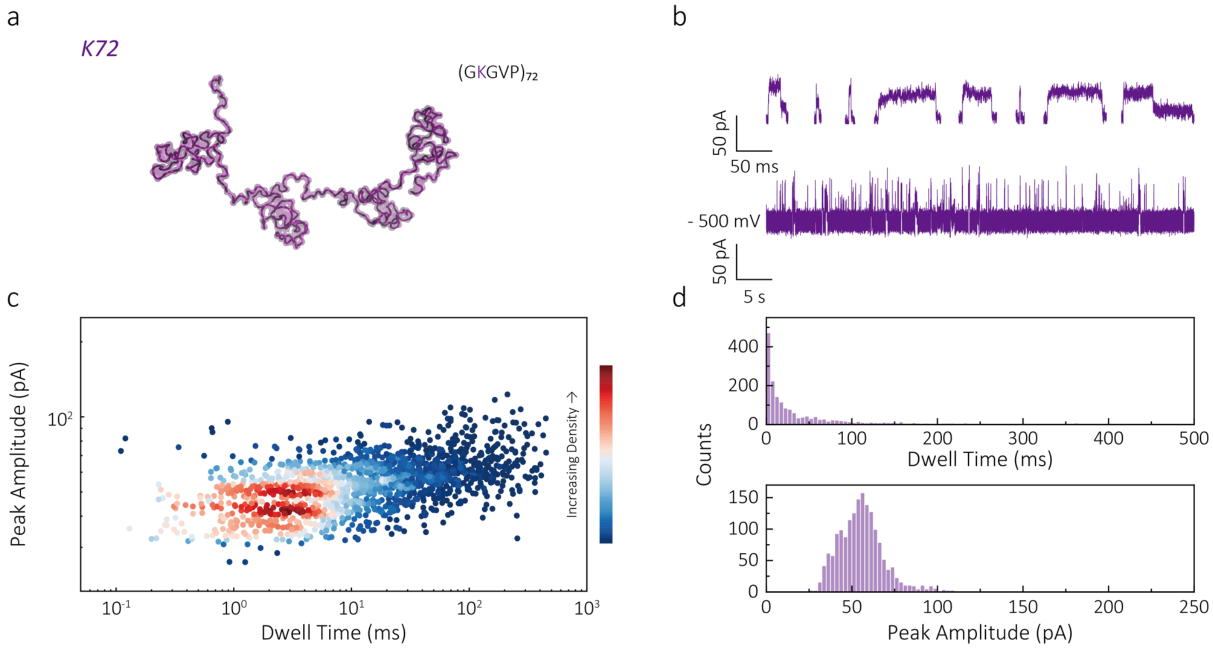

Figure S11. Nanopore detection of K72. (a) Protein model of K72 predicted by I-TASSER.[2] (b) Typical events and current-time traces of 1 nM K72 translocation recorded in 1 M KCl, 10 mM Tris-EDTA, pH 8.0 buffer at -500 mV. Data were sampled at 1 MHz and digitally filtered using a Bessel low-pass filter at 10 kHz. (c) Scatter plot of peak amplitude versus dwell time. (d) Distribution of the dwell time and peak amplitude for K72 translocation.

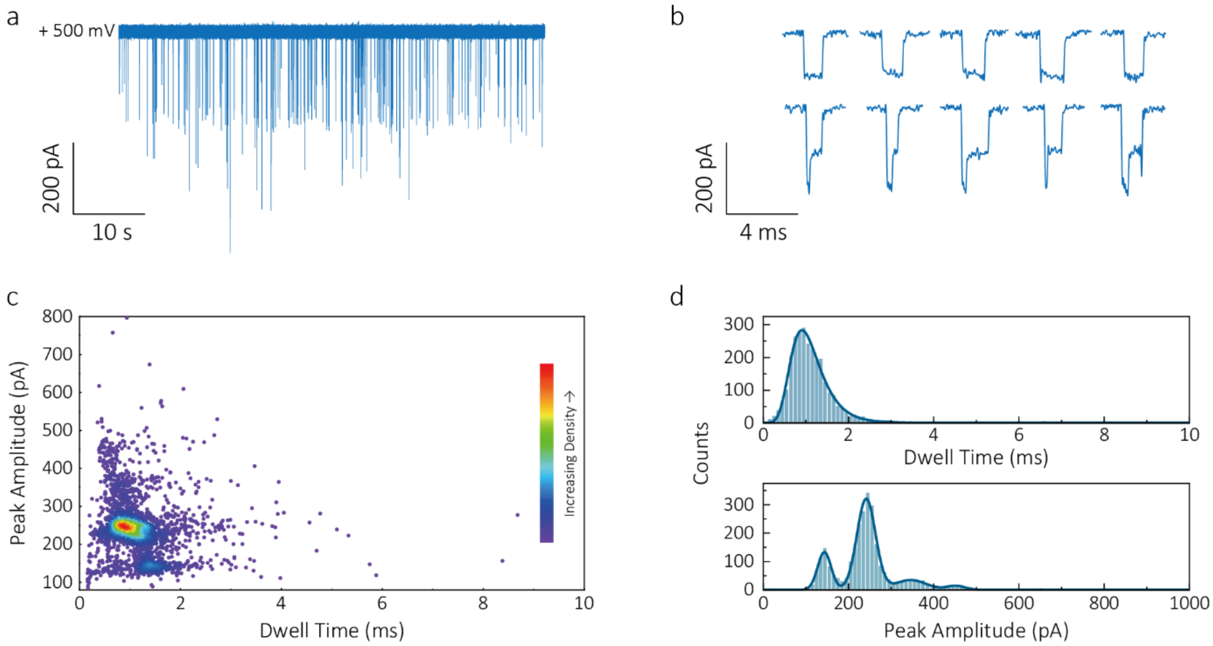

Figure S12. Nanopore detection of lambda-DNA. (a) Representative current-time traces of 100 pM lambda-DNA translocation were recorded in 1 M KCl, 10 mM Tris-EDTA, pH 8.0 buffer at +500 mV. Data were sampled at 1 MHz and digitally filtered using a Bessel low-pass filter at 10 kHz. (b) Typical events of unfolded and folded DNA molecules. (c) Scatter plot of peak amplitude versus dwell time. (d) Distribution of the dwell time and peak amplitude for lambda-DNA translocation showing multiple folding levels.

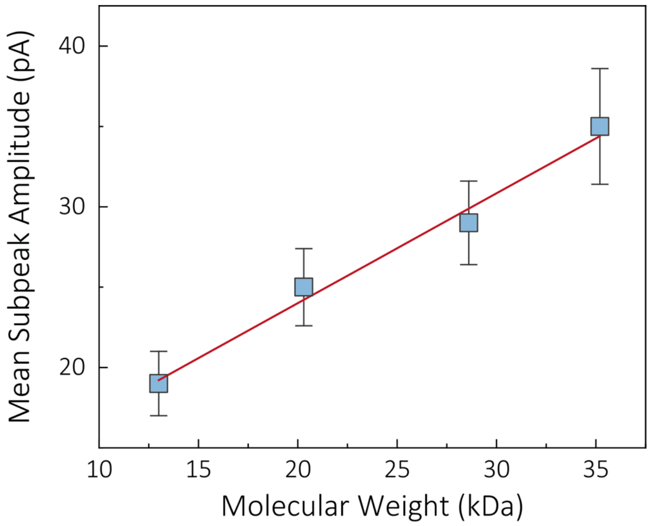

Figure S13. Dependence of subpeak amplitude on molecular weight. Subpeak amplitude of different proteins as a function of molecular weight. The red line represents a linear fitting through the blue markers. This experiment was repeated at least three times for each protein, showing a similar linear dependence for these globular proteins.

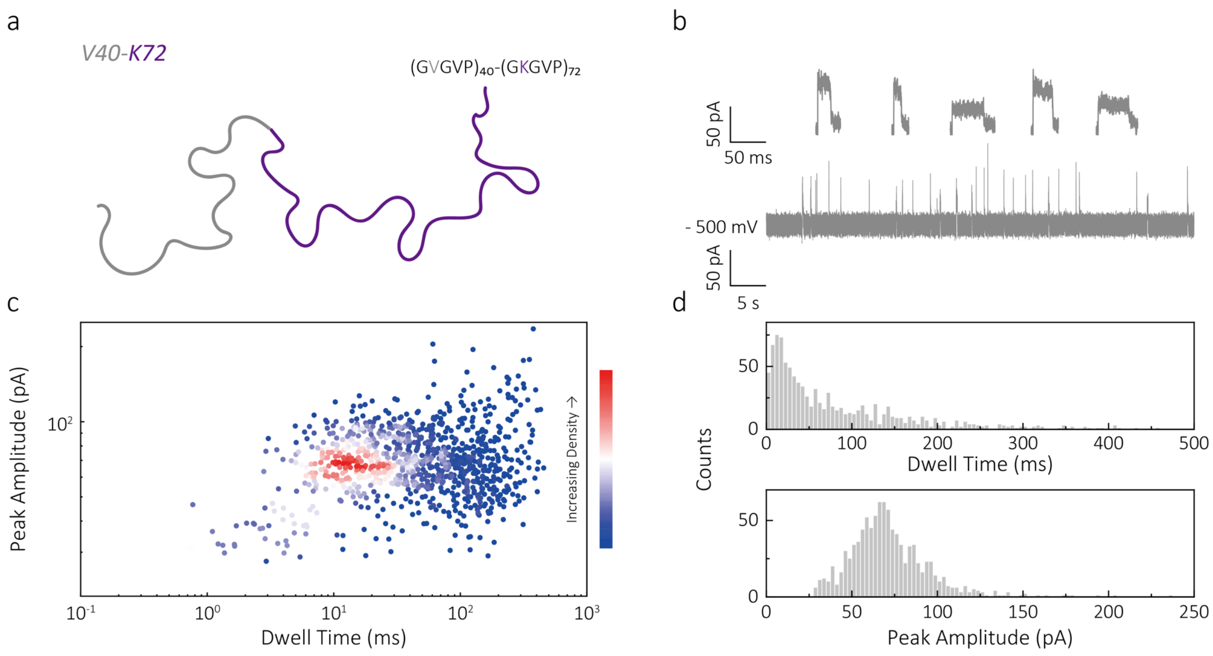

Figure S14. Nanopore detection of V40K72. (a) Protein model and sequence of V40K72. (b) Typical events and current-time traces of 1 nM V40K72 translocation were recorded in 1 M KCl, 10 mM Tris-EDTA, pH 8.0 buffer at -500 mV. Data were sampled at 1 MHz and digitally filtered using a Bessel low-pass filter at 10 kHz. (c) Scatter plot of peak amplitude versus dwell time. (d) Distribution of the dwell time and peak amplitude for V40K72 translocation.

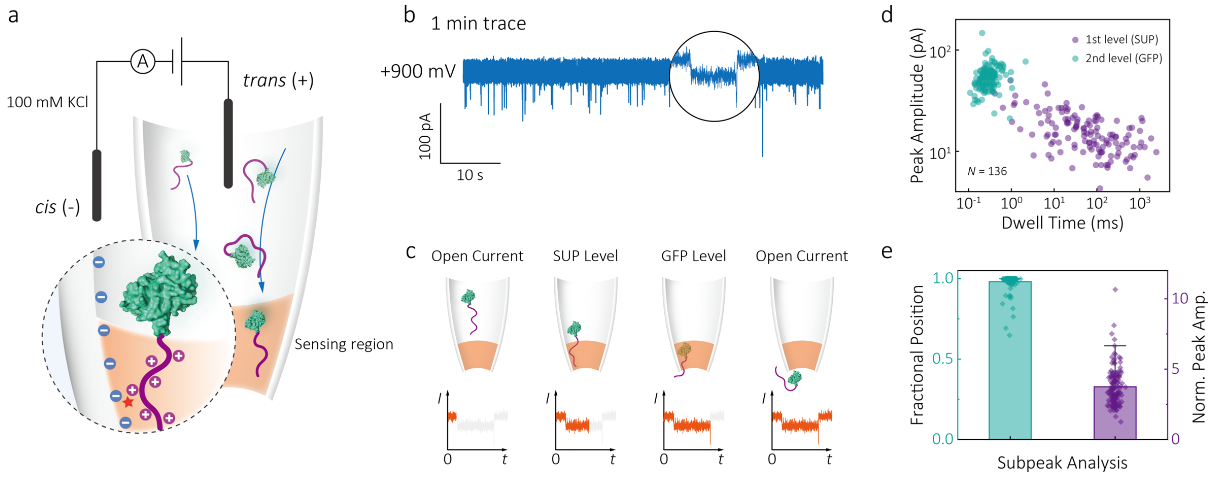

Figure S15. Nanopore detection of eGFP-K72 at low ionic strengths. (a) Schematic of eGFP-K72 translocation at a low salt concentration (100 mM KCl, 10 mM10 mM Tris-EDTA, pH 8.0 buffer). In this case, the protein was added inside the nanopipette (*trans* chamber), and a negative voltage was applied to drive protein molecules translocating from *trans* to *cis* side. Cationic SUPs had stronger electrostatic interaction with the nanopore inner walls at low ionic strengths. (b) Typical events and current-time traces of 10 nM eGFP-K72 show a two-level current signature with a subpeak at the end. (c) Illustration of eGFP-K72 translocation and corresponding current blockade. (d) Scatter plots of peak amplitude versus dwell time for the first level (SUP level) and second level (GFP level). (e) Statistics for the subpeak position and subpeak amplitude.

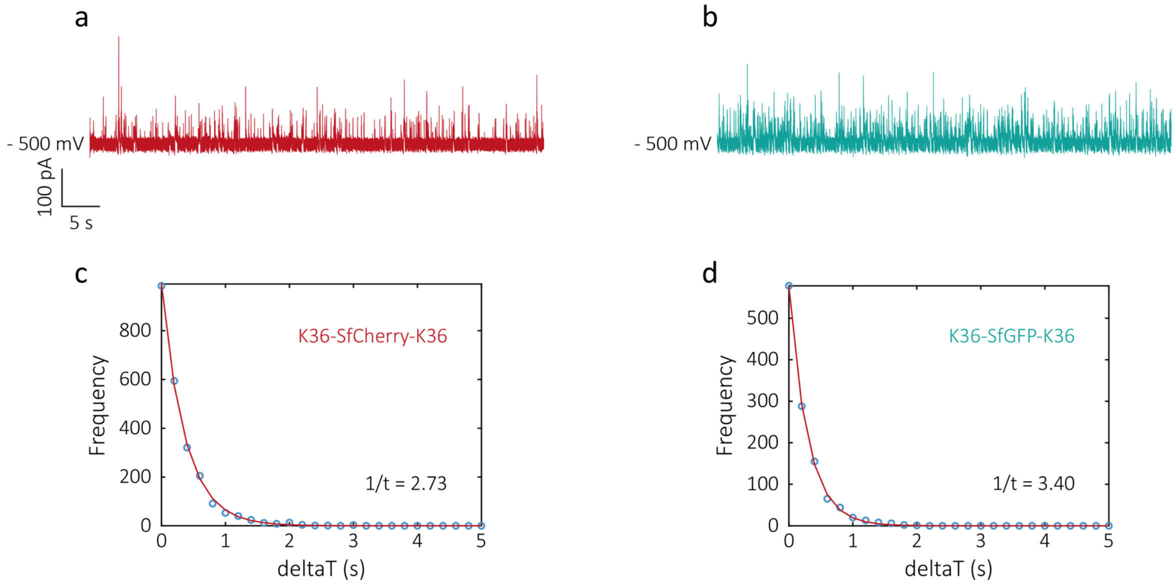

Figure S16. Nanopore detection of K36-SfCherry-K36 and K36-SfGFP-K36. Representative current-time traces of (a)1 nM K36-SfCherry-K36 and (b) 1 nM K36-SfGFP-K36 translocation recorded in 1 M KCl, 10 mM Tris-EDTA, pH 8.0 buffer at -500 mV. Data were sampled at 1 MHz and digitally filtered using a Bessel low-pass filter at 10 kHz. Distributions of the elapsed time between successive captured events for (c) 1 nM K36-SfCherry-K36 and (d) 1 nM K36-SfGFP-K36 with a single-exponential decay fitting.

Section S4. Quantification of single protein-protein interactions using protein-SUP probes

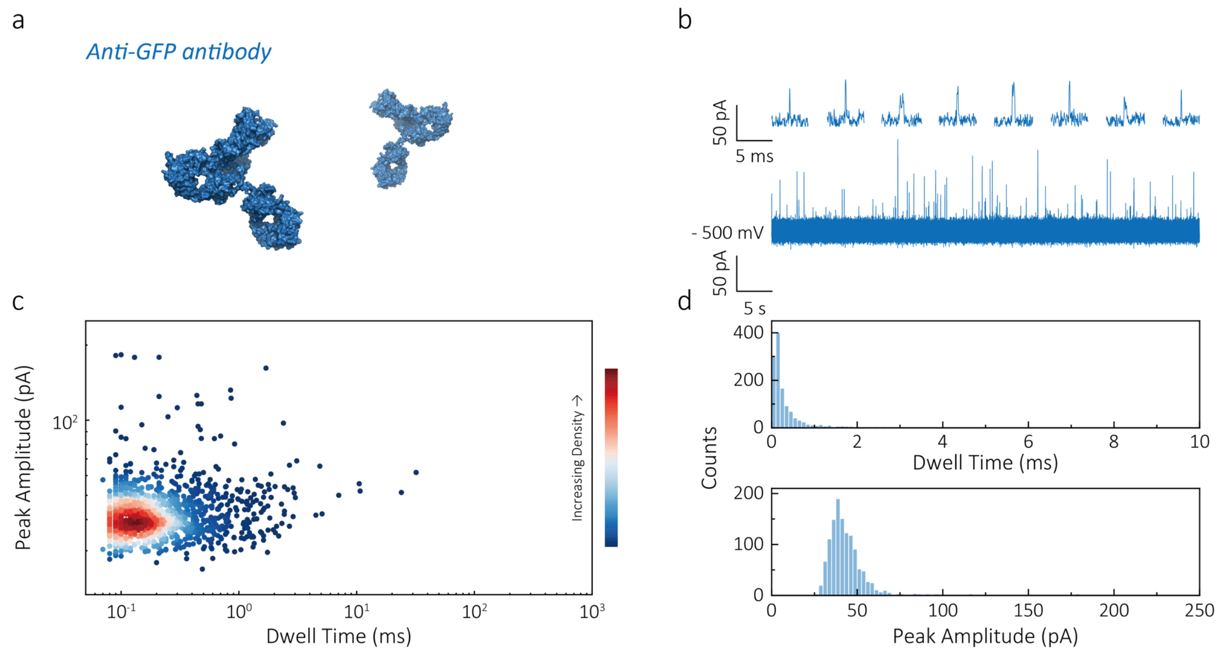

Figure S17. Nanopore detection of anti-GFP antibody. (a) Protein model of anti-GFP antibody (IgG1). (b) Typical events and current-time traces of 10 nM anti-GFP antibody translocation recorded in 1 M KCl, 10 mM Tris-EDTA, pH 8.0 buffer at -500 mV. Data were sampled at 1 MHz and digitally filtered using a Bessel low-pass filter at 10 kHz. (c) Scatter plot of peak amplitude versus dwell time. (d) Distribution of the dwell time and peak amplitude for anti-GFP antibody translocation.

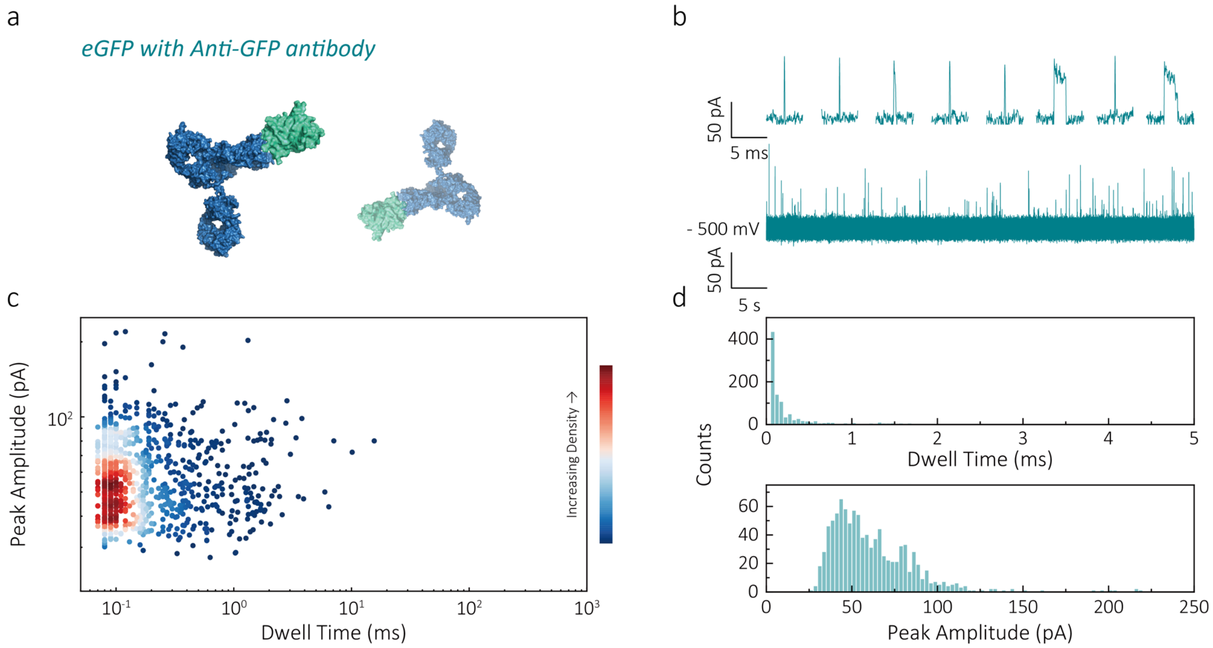

Figure S18. Nanopore detection of eGFP with anti-GFP antibody. (a) Protein model of eGFP-anti-GFP antibody complex. (b) Typical events and current-time traces of 1 nM eGFP + 10 nM anti-GFP antibody translocation were recorded in 1 M KCl, 10 mM Tris-EDTA, pH 8.0 buffer at -500 mV. Data were sampled at 1 MHz and digitally filtered using a Bessel low-pass filter at 10 kHz. (c) Scatter plot of peak amplitude versus dwell time. (d) Distribution of the dwell time and peak amplitude for the translocation of eGFP with anti-GFP antibody (1: 10).

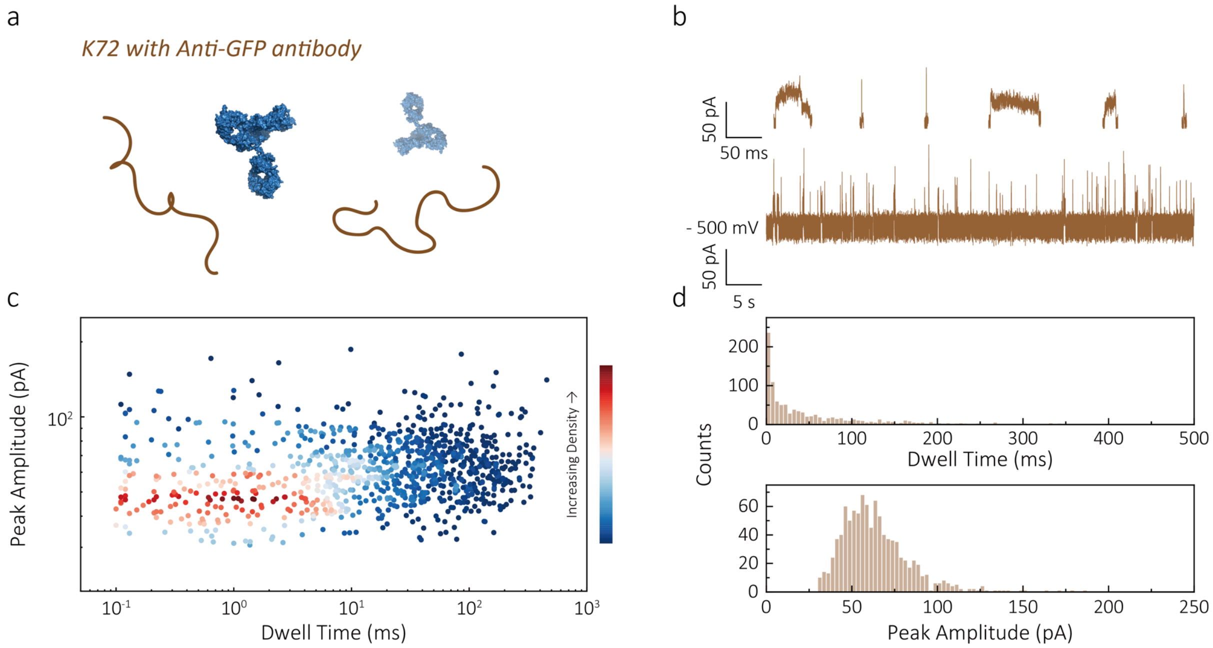

Figure S19. Nanopore detection of K72 with anti-GFP antibody. (a) Protein model of K72 and anti-GFP antibody. (b) Typical events and current-time traces of 1 nM K72 + 10 nM anti-GFP antibody translocation were recorded in 1 M KCl, 10 mM Tris-EDTA, pH 8.0 buffer at -500 mV. Data were sampled at 1 MHz and digitally filtered using a Bessel low-pass filter at 10 kHz. (c) Scatter plot of peak amplitude versus dwell time. (d) Distribution of the dwell time and peak amplitude for the translocation of K72 with anti-GFP antibody (1: 10).

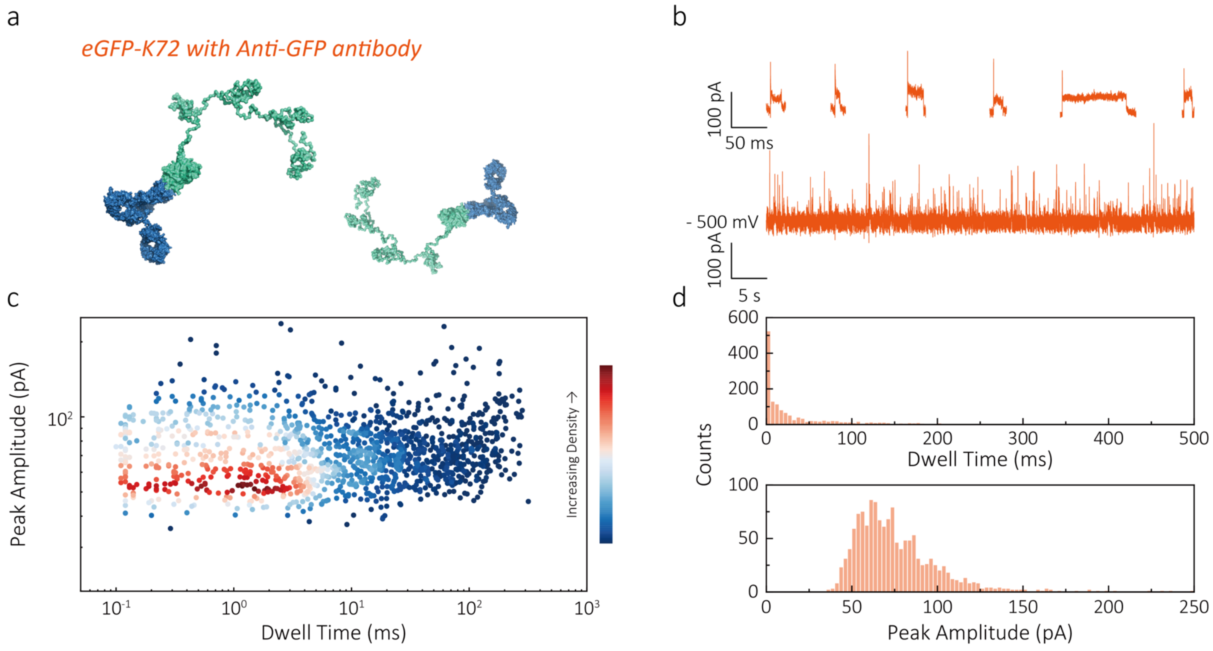

Figure S20. Nanopore detection of eGFP-K72 with anti-GFP antibody. (a) Protein model of eGFP-K72-anti-GFP antibody complex. (b) Typical events and current-time traces of 1 nM eGFP-K72 + 10 nM anti-GFP antibody translocation were recorded in 1 M KCl, 10 mM Tris-EDTA, pH 8.0 buffer at -500 mV. Data were sampled at 1 MHz and further low-pass filtered at 10 kHz. (c) Scatter plot of peak amplitude versus dwell time. (d) Distribution of the dwell time and peak amplitude for the translocation of eGFP-K72 with anti-GFP antibody (1: 10).

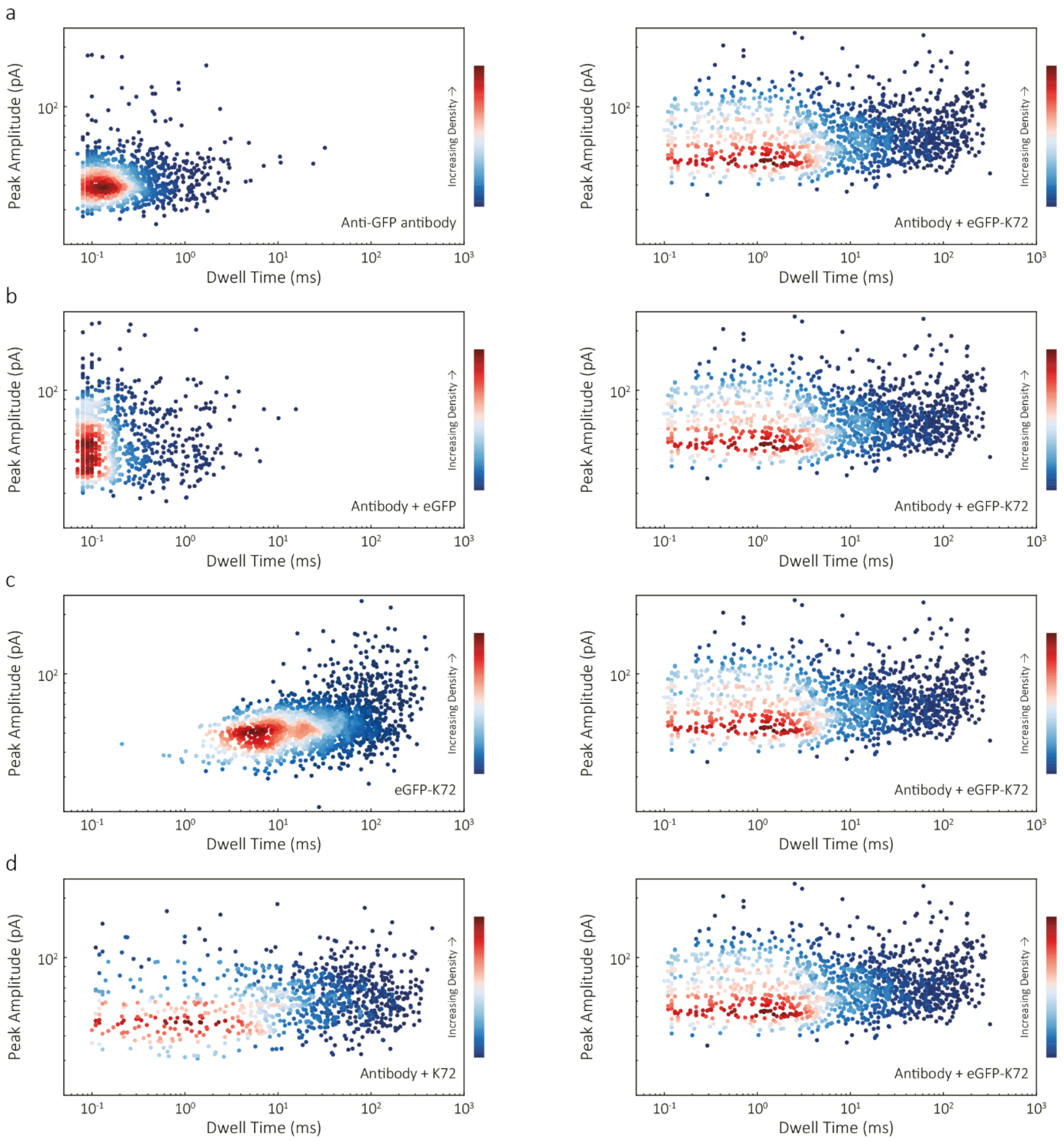

Figure S21. Identification of protein-protein interactions with SUP and without SUP. Scatter plots of (a) 10 nM anti-GFP antibody, (b) 1 nM eGFP + 10 nM anti-GFP antibody, (c) 1 nM eGFP-K72 and (d) 1 nM K72 + 10 nM anti-GFP antibody was compared with 1 nM eGFP-K72 + 10 nM anti-GFP antibody.

Section S5. Data analysis

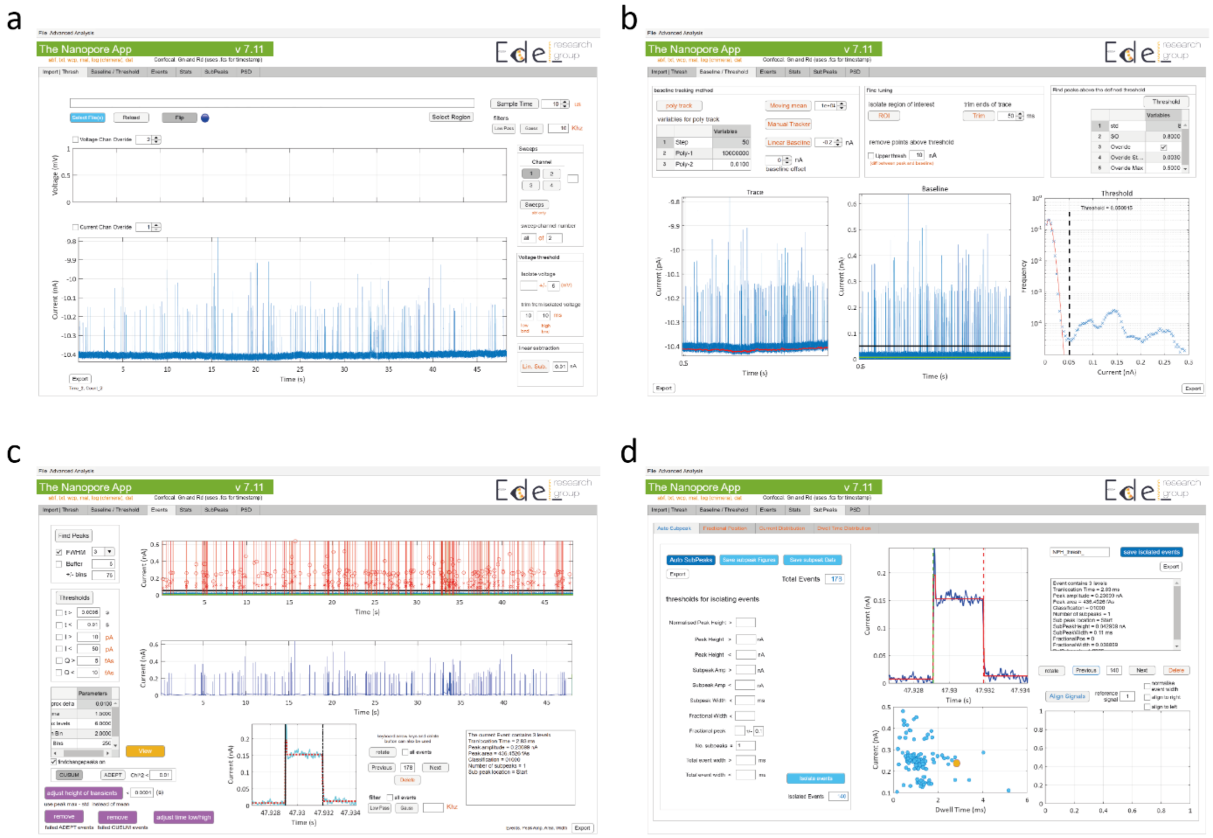

Figure S22. Workflow of the Nanopore App for data analysis. (a) Data import and filter. In this step, nanopore data was loaded into the Nanopore App and then resampled and low-pass filtered dependent on the signal-to-noise ratio (e.g., 1 MHz sampling rate and 10 kHz low-pass filter). (b) Baseline tracking and threshold. The baseline was tracked to compensate for fluctuations in the current recordings. A histogram of all points was fitted by a Poisson distribution for the baseline, and a threshold (e.g., 7 σ) was used to classify events. (c) Peak selection. Events with a peak amplitude above the set threshold were selected. (d) Subpeak analysis. Multiple levels of an event were classified using cumulative sum (CUSUM) analysis, and subpeak amplitude and fractional position could be exported.

References

(1) Perry, D.; Momotenko, D.; Lazenby, R. A.; Kang, M.; Unwin, P. R. Characterization of Nanopipettes. *Anal Chem* **2016**, *88* (10), 5523–5530. https://doi.org/10.1021/acs.analchem.6b01095.

(2) Yang, J.; Yan, R.; Roy, A.; Xu, D.; Poisson, J.; Zhang, Y. The I-TASSER Suite: Protein Structure and Function Prediction. *Nature Methods*. Nature Publishing Group January 1, 2014, pp 7–8. https://doi.org/10.1038/nmeth.3213.
